# Explainable AI reveals changes in skin microbiome composition linked to phenotypic differences

**DOI:** 10.1038/s41598-021-83922-6

**Published:** 2021-02-25

**Authors:** Anna Paola Carrieri, Niina Haiminen, Sean Maudsley-Barton, Laura-Jayne Gardiner, Barry Murphy, Andrew E. Mayes, Sarah Paterson, Sally Grimshaw, Martyn Winn, Cameron Shand, Panagiotis Hadjidoukas, Will P. M. Rowe, Stacy Hawkins, Ashley MacGuire-Flanagan, Jane Tazzioli, John G. Kenny, Laxmi Parida, Michael Hoptroff, Edward O. Pyzer-Knapp

**Affiliations:** 1 The Hartree Centre, Sci-Tech Daresbury, IBM Research, Daresbury, WA4 4AD UK; 2 T.J. Watson Research Center, IBM Research, Yorktown Heights, NY 10598 USA; 3 Unilever Research & Development, Port Sunlight, CH63 3JW UK; 4 Unilever Research and Development, Sharnbrook, MK44 1LQ UK; 5 Scientific Computing Department, STFC Daresbury Lab, Daresbury, WA4 4AD UK; 6 IBM Research - Zurich, Saumerstrasse 4, 8803 Rueschlikon, Switzerland; 7 University of Birmingham, Birmingham, UK; 8 Unilever Research & Development, Trumbull, CT 06611 USA; 9 Institute of Integrative Biology, The University of Liverpool, The Bioscience Building, Liverpool, L697ZB UK; 10 Department of Computing and Mathematics, Manchester Metropolitan University (MUU), Manchester, M15 6BH UK; 11 Department of Computer Science, University of Manchester (UoM), Manchester, M13 9LP UK

**Keywords:** Classification and taxonomy, Data processing, Machine learning, Statistical methods, Metagenomics, Microbiome

## Abstract

Alterations in the human microbiome have been observed in a variety of conditions such as asthma, gingivitis, dermatitis and cancer, and much remains to be learned about the links between the microbiome and human health. The fusion of artificial intelligence with rich microbiome datasets can offer an improved understanding of the microbiome’s role in human health. To gain actionable insights it is essential to consider both the predictive power and the transparency of the models by providing explanations for the predictions. We combine the collection of leg skin microbiome samples from two healthy cohorts of women with the application of an *explainable artificial intelligence (EAI)* approach that provides accurate predictions of phenotypes with explanations. The explanations are expressed in terms of variations in the relative abundance of key microbes that drive the predictions. We predict skin hydration, subject's age, pre/post-menopausal status and smoking status from the leg skin microbiome. The changes in microbial composition linked to skin hydration can accelerate the development of personalized treatments for healthy skin, while those associated with age may offer insights into the skin aging process. The leg microbiome signatures associated with smoking and menopausal status are consistent with previous findings from oral/respiratory tract microbiomes and vaginal/gut microbiomes respectively. This suggests that easily accessible microbiome samples could be used to investigate health-related phenotypes, offering potential for non-invasive diagnosis and condition monitoring. Our EAI approach sets the stage for new work focused on understanding the complex relationships between microbial communities and phenotypes. Our approach can be applied to predict any condition from microbiome samples and has the potential to accelerate the development of microbiome-based personalized therapeutics and non-invasive diagnostics.

## Introduction

The associations between the human microbiome and individual health-related phenotypes are increasingly being studied, giving rise to intervention strategies including prebiotics and probiotics^1^ and personalized therapies^2^. An improved understanding of how microbial taxa contribute to health and wellbeing, coupled with an increasing ability to characterize the microbiome, can drive and accelerate the development of personalized microbiome-based treatments. As microbiome research expands and as sequencing technologies continue to advance, the volume and complexity of the data collected inexorably increases. Therefore, the necessity to develop sophisticated methods for analyzing microbiome data to derive actionable insights becomes increasingly important. Machine learning (ML) has the potential for building predictive models that can provide powerful comprehension of the complex interactions between microbial communities and their host organisms^3^. ML has been applied to microbiome data to investigate several important questions regarding the clustering of microbial species, taxonomic assignment, comparative metagenomics and gene prediction.

Recently, a number of studies have been published on phenotype prediction from microbiome data^4–10^, including some particularly focused on identifying discriminatory microbial taxa^11^ or microbial signatures^12^. As ML is becoming increasingly deployed in patient-relevant settings, it is essential to consider both the predictive power of the models and the transparency of the recommendations by providing an explanation for the predictions^13^. This is often referred to as the *explainability* of the model. Interpretable AI/ML for microbiome data is increasingly applied since explanations of the predictions are needed^14–16^. However, more can be done in terms of explaining the mechanisms of the ML algorithm predictions for microbiome data.

We propose an *explainable artificial intelligence* (EAI) approach to identify key predictive taxa and to investigate how the distributions of these microbial taxa drive the prediction of different phenotypic values. The explanations are expressed in terms of key variations in microbiome composition. Our streamlined approach includes three machine learning models—random forest RF^17^, XGBoost^18^ and LightGBM^19^—to predict the phenotypes. Each model is tuned using the training dataset to predict the host phenotype, for the different classification and regression tasks. Once tuned and trained, the optimized models are applied to predict unseen samples in the test dataset. Performances on the test set and cross validation are compared based on the lowest Mean Absolute Error for regression tasks or highest accuracy (given by F1-score, precision and recall metrics) for classification tasks. The best model is then selected based on the performance scores, balancing the best score with the least difference in score between the training and the test datasets (i.e., least overfitting). The explanations of the best model are then provided using an explainable AI algorithm called SHapley Additive exPlanations (SHAP)^20^. A detailed discussion of our EAI approach can be found in the Methods section.

To exemplify the power of explainable AI as a means to derive actionable insights from complex interactions between microbes and their hosts, this study is focused on the human skin microbiome. The technical ease of acquiring skin microbiome datasets—it is reliant only upon swabbing or scrubbing of skin to collect samples—and the recent increase in the interest in analyzing the human skin microbiome^21–25^ makes skin an appealing target. The human skin is a large, heterogeneous organ that protects the body from pathogens while supporting microorganisms that influence human health and disease^26^.

For this study, a total of 1200 time-series leg skin microbiome samples (bacterial 16S rRNA gene sequencing) as well as associated skin hydration measures (visual assessment, pH, conductance, capacitance) were collected from 62 Canadian women (21–65 years of age) displaying healthy (non-dry) or moderately dry skin. The time series samples were collected between April and July 2017. Supplementary Figure 1 shows the number of samples per subject for the Canada cohort. Additional phenotypic data (i.e., age, smoking habits and menopausal status) were obtained from subject questionnaires. This study is primarily focused on the Canada cohort. However, to investigate the generalizability of the EAI approach we used a second independent UK cohort of 278 samples from 102 women. The UK cohort serves as a true hold-out test set to predict the skin hydration with the ML models trained on the Canada cohort. The menopausal and smoking status metadata were not available for the UK cohort. Table 1 reports the distribution of the four phenotypes for the Canada cohort and of skin hydration for the UK cohort (see the Methods section and the Clinical design section in Supplementary Information for more detail). The microbial features used as input to our EAI approach, are derived from the observed genera, i.e., Operational Taxonomic Units (OTUs) classified to genus level. Therefore, we use the terms *genera* and *features* interchangeably. Relative abundances of 186 genera were obtained from taxonomic analysis of the sequenced reads from both studies.

**Table 1:** Metadata for Canada and UK cohorts.

Time series Samples	Country	Sex	Corneometer	Age	Menopausal status	Smoking status
1200 samples of 62 subjects	Canada	Female	[2.4–57.2] Median ~ 24.46 Mean ~ 24.36 Std ~ 8.87	[21–65] Median ~ 56.0 Mean ~ 52.7 Std ~ 11.36	324 samples of 18 subjects in pre-menopause 876 of 44 subjects in post-menopausal	286 samples of 19 smokers 914 samples of 43 non-smokers
278 samples of 102 subjects	UK	Female	[8.1–78.4] Median ~ 27.3 Mean ~ 31.70 Std ~ 12.42	[19–55] Median ~ 39.5 Mean ~ 38.74 Std ~ 9.09	–	–

See Supplementary Figure 1 for more details on the distributions of corneometer, age and samples per subjects. Also see Methods and Supplementary Notes for more detail on the sample collection.

Our EAI approach uses the microbiome-derived features to predict skin hydration, age, menopausal and smoking status for different subsets of the Canada cohort; the first 62 samples taken from each subject, the last 62 samples taken from each subject, and the time series samples blocked by subject (this ensures that samples from the same subject are not present both in the training and test datasets when tuning, training and evaluating our ML models). For each phenotype, we then compared the predictive performance of the ML models when using 62 “first samples taken”, 62 “last samples taken” and 1200 samples “blocked by subject”. Similarly, we applied our EAI approach to predict the skin hydration of two different subsets of the UK cohort; the 102 first samples taken from each subject and the 278 time series samples blocked by individual. For the best model and the best subset, we then inferred, through the model’s explanation, how the most impactful microbial genera are contributing to the prediction of each phenotype.

We decided to investigate the four phenotypes (skin hydration, age, menopausal and smoking status) for the following reasons. Firstly, there is an increasing recognition of microbiome dysbiosis as a factor in atopic skin that is linked to psoriasis, eczema, acne, dermatitis and other disorders^25, 27–29^. Previous studies characterized microbiome signatures of different sites highlighting topographical and temporal variance in the microbiome composition across dry, moist and sebaceous skin sites^26, 30–32^. However, there is still a limited understanding of changes in the microbiome associated with cosmetic dry skin. We show that our EAI approach has the potential to provide insights on the effect of skin care and hygiene products on the molecular and microbiome composition of the skin^33^. Moreover, being able to infer key compositional changes in the skin microbiome associated with age may offer insights into the aging process that could be used to develop products that counteract skin aging. Although a natural part of aging, the onset of menopause in women is a highly significant life event, especially for those women for whom the onset occurs earlier than expected. Menopause is currently diagnosed based on the symptoms, but a blood test to measure the hormone levels may be carried out for younger women. The power of predicting the onset of menopause through a simple scrub of the leg could be transformational to many women, as it points to the potential for a non-invasive diagnostic tool to be used as a condition monitor. Finally, previous studies have highlighted the strong impact of smoking on gastrointestinal microbiota^34, 35^, and the differences in microbial community composition of the upper respiratory tract between healthy smokers and non-smokers^36^. Although smoking is considered a contributing factor to skin aging and systemic health^37^, its potential to influence the skin microbiome has not been fully investigated.

Through the application of our carefully calibrated EAI approach we were able to accurately predict the four phenotypes for the first samples taken from each subject of the Canada cohort and we identified skin microbial signatures associated with diverging values of each phenotype. Our findings demonstrate the potential of explainable artificial intelligence in contributing to the growing body of knowledge of host-microbe interactions, and its future impact in many research fields, from cosmetic and medical research to forensic science and personal health monitoring^29, 33, 38, 39^.

## Results

**Figure 1:**
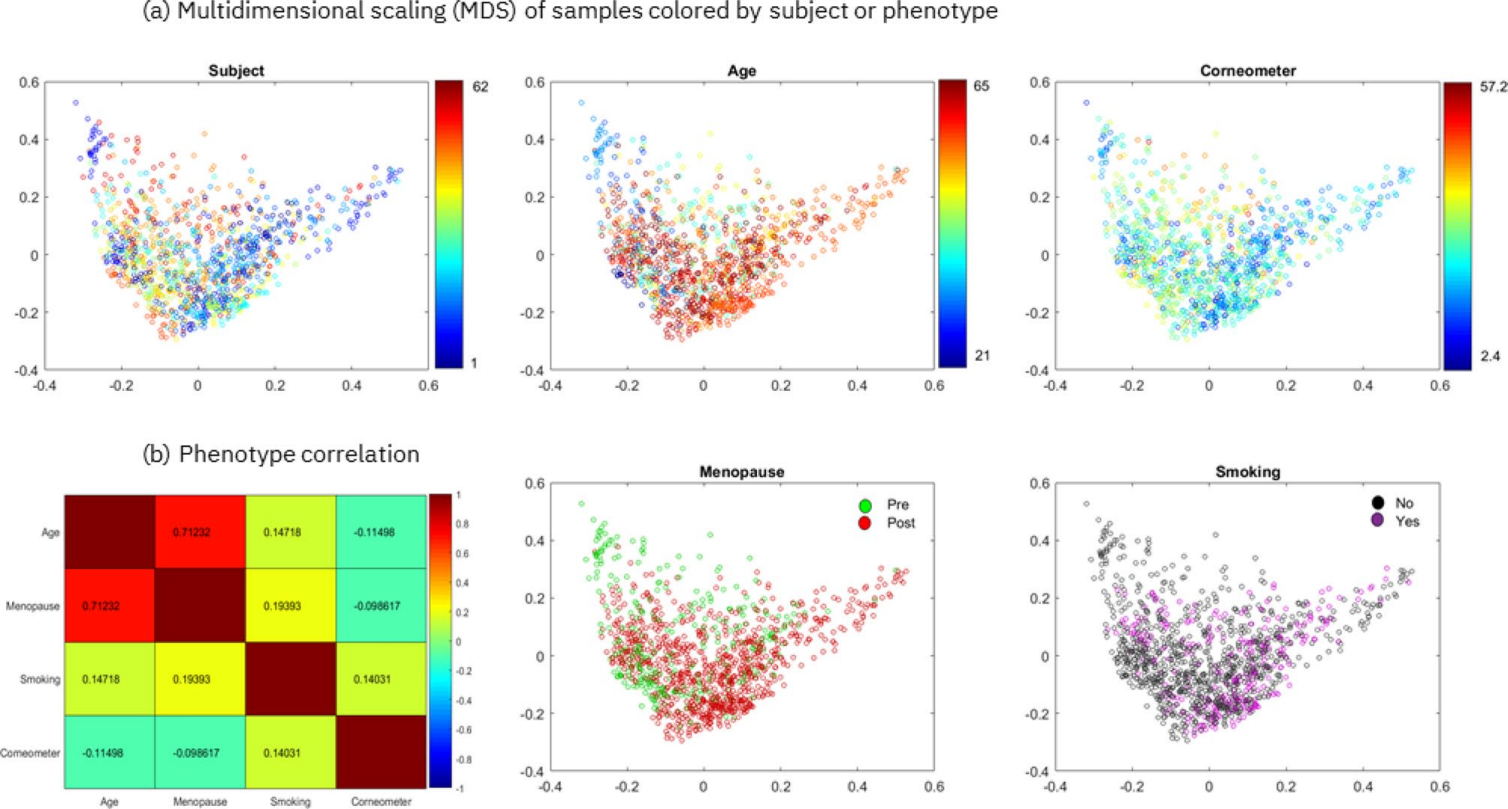
Multidimensional scaling (MDS) and phenotype correlation for the Canada cohort. (**a**) Multidimensional scaling of pairwise Bray–Curtis dissimilarities of 1200 samples colored by subject (the 62 subjects are each represented by a randomly selected color) or by phenotypic value. (**b**) Pairwise Spearman’s correlation is shown between each pair of phenotypes, except point-biserial correlation is shown when comparing binary vs. continuous phenotypes (the encoding for computing the correlation for the binary phenotypes is the following: smoking no/yes = 0/1, menopause pre/post = 0/1). The first two eigenvalues of the MDS represent 12% and 10% of the sum of all positive eigenvalues. This image has been generated using Matlab version R2017a at https://www.mathworks.com/products/matlab.html.

In the following sections we present the results obtained when we predicted skin hydration, age, menopausal status and smoking status using the Canada cohort. To account for possible confounding factors when predicting the four phenotypes, we checked if the multidimensional projection of pairwise Bray–Curtis dissimilarities of 1200 samples showed biases or separability of the samples by subject and by phenotype (Fig. 1a). The results indicate that the samples are centered with no clear trends in either dimension of the two-dimensional scaling and that they are not separable by subject, age or corneometer measurement. Similarly, Supplementary Figure 5a reports the multidimensional projection of the Bray–Curtis dissimilarities of the 62 first samples taken from the Canada cohort, showing that the 62 samples are not separable by subject or by any of the phenotypes.

Moreover, we examined the connections between the skin hydration (corneometer measurement), age, menopause and smoking phenotypes by computing their pairwise correlations (Fig. 1b). Only age and menopause were found to be strongly correlated (~ 0.71 point-biserial correlation)), as expected. To address this, we considered possible confounding factors when predicting age and menopause and showed that we were able to predict age within the pre- and post-menopausal groups separately (Supplementary Table 3). Also, in section “Leg skin microbiome accurately predicts menopausal status” we discuss the differences in predictive genera between the age and menopause models and the differences in impactful genera, that are predictive of age, between the pre-menopausal and post-menopausal groups. With these checks in place, we proceeded with phenotype prediction.

Firstly, to avoid potential overfitting that could provide over-optimistic predictive performances when using time-series samples, we blocked the analysis by subject (for both the Canada and UK cohorts). This ensured that the samples from the same subjects were not present both in the test and training datasets during tuning, training and cross validation of the ML models. Secondly, to take into account repeated-measurement effects, we applied our EAI approach on two different subsets of 62 Canadian samples (one sample per subject). More precisely, the first subset contained the first sample taken from each subject (i.e., at the first time point), while the second subset contained the last sample taken from each subject. For training and testing the ML models we randomly selected the 80% and 20% of the samples in each subset.

For each phenotype, we compared the predictive performance of three fine-tuned ML models on the different subsets (see Supplementary Tables 1 and 2 for the full list of results). Tables 2 and 3 show summaries of the predictive performances of the best selected models that have been tuned, trained and cross validated either on the 62 first samples taken from the 62 Canada subjects or on the 1200 time series samples of the Canada cohort blocked by subject. Overall, we found that the most predictive subset for the Canada cohort encompassed the 62 first samples taken. In fact, the decrease in predictive performance when using time series samples blocked by subjects is likely due to the use of multiple samples per subject that leads to overfitting to intra-personal characteristics.

**Table 2:** Summary of predictive performance of the best ML models for regression tasks.

Phenotype	Dataset	Model	MAE Test	MAE Train	Mean MAE CV	Std MAE CV
Corneometer	Canada—62 first samples taken	RF	5.54	4.58	5.70	0.46
	Canada—1200 samples blocked by individual	LightGBM	7.34	1.68	5.09	0.24
	UK—102 first samples taken	RF	11.8	4.61	11.85	2.07
	UK—278 samples blocked by individual	LightGBM	8.96	7.53	9.12	0.87
Age	Canada—62 first samples taken	RF	6.38	6.73	9.69	0.87
	Canada—1200 samples blocked by individual	RF	10.78	0.38	9.19	2.11

Three ML models (RF, LightGBM, XGboost) have been evaluated on different subsets of the Canada cohort (62 samples taken from each subject at the first time point, and 1200 time series samples blocked by subject) and the UK cohort (102 samples taken from each subject at the first time point, and 278 times series samples blocked by subject). When applied to time series samples, the ML models have been tuned and trained blocking by individual, e.g., samples of the same subjects are not present both in the training and test datasets. The table reports the performances (mean absolute error (MAE) on training, testing and cross validation) of the best fine-tuned model per dataset and phenotype. Supplementary Table 1 shows the full list of predictive performances of all the ML models per dataset and phenotype.

**Table 3:** Summary of predictive performance of the best ML models for classification tasks.

Phenotype	Dataset	Model	F1 score per class Test	F1 score Test	F1 score Train	Precision per class Test	Recall per class Test	Ave F1-score CV	Std F1-score CV
Menopausal status	62 first samples	LightGBM	[0.86, 0.95]	0.92	0.98	[1., 0.9]	[0.75, 1. ]	0.93	0.06
	1200 samples blocked by individual	XGBoost	[0.89, 0.75]	0.85	1	[0.89, 0.75]	[0.89, 0.75]	0.82	0.07
Smoking status	62 first samples	XGBoost	[0.89, 0.75]	0.85	0.98	[0.89, 0.75]	[0.89, 0.75]	0.72	0.12
	1200 samples blocked by individual	LightGBM	[0.88, 0.10]	0.74	1.0	[0.82, 0.21]	[0.94, 0.07]	0.93	0.08

Three ML models (RF, LightGBM, XGboost) have been evaluated on different subsets of the Canada cohort (62 samples taken from each subject at the first time point, and 1200 time series samples blocked by individual). When applied to time series samples, the ML models have been tuned and trained blocking by individual, e.g., samples of the same subjects are not present both in the training and test datasets. The table reports F1-score, precision and recall per class as computed on the test dataset, weighted average F1-score on the test, training datasets and on cross validation. The table reports the performances scores of the best fine-tuned model per dataset and phenotype, while Supplementary Table 2 shows the full list.

**Figure 2:**
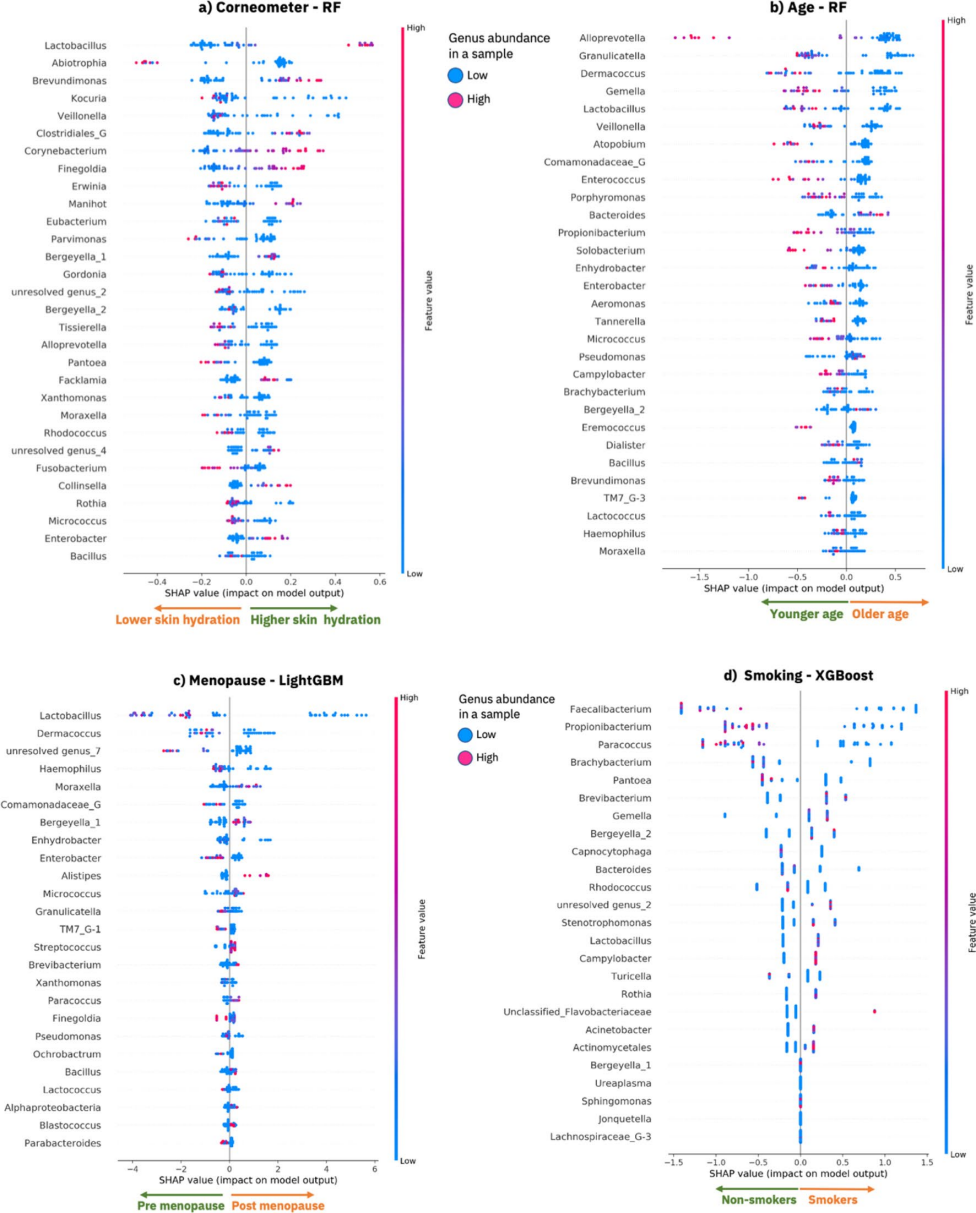
Model explanations for the 62 first samples taken from the Canada cohort. SHAP summary dot plot as computed by SHAP using the best optimized ML model that has been trained on 80% of the training data. Each plot provides an overview of which features are most important for a model and visualizes how the value of each feature (i.e., the genus abundance in the samples) contributes, either positively or negatively, to the prediction of phenotypic values; (**a**) lower or higher corneometer measurements, (**b**) lower or higher values of age, (**c**) pre-menopausal or post-menopausal status and (**d**) non-smokers or smokers. The features are sorted by the sum of the absolute SHAP values over all the samples in the training dataset. Each dot is a sample, and its color represents a feature value (i.e., genus abundance) for the sample. Red dots are samples for which a genus (row) is enriched, while blue dots are samples for which a genus is lower in abundance. Clusters of red samples on the right side of the x-axis means that the genus is abundant in those samples and it is contributing to the prediction of a higher phenotypic value (indicated by the x-axis annotation of arrows pointing right). Clusters of red samples (dots) on the left side of the y-axis means that the genus is enriched for those samples and it is contributing to the prediction of a lower phenotypic value for those samples (indicated by the x-axis annotation of arrows pointing left). This image was created using SHAP^20^ version 0.34.0 (https://github.com/slundberg/shap).

Finally, for each predicted phenotype we generated the explanations of the best model (i.e., the model that overall provided the best predictive performance and insights) for both the training and the test datasets (Fig. 2 and Supplementary Figure 6 respectively) that we discuss in the following sections.

### Explainable skin hydration model identifies key microbes predictive of skin hydration

We investigated the prediction of both individual and combined measures of skin hydration, and found that corneometer measurement, a widely used and robust method for assessing skin hydration^39^, was the most appropriate measure for the Canada study (see section Phenotype analysis in Supplementary Information and Supplementary Figures 2–4). Efforts to enrich the corneometer score with other measures did not provide sufficient additional improvement in predictive performance as to merit their inclusion. Higher corneometer score values correspond to higher skin hydration; thus, we adopt the terms corneometer score and skin hydration interchangeably.

When examining the relationship between corneometer and variation in skin microbiome composition, the best selected model was the optimized RF that was tuned, trained and evaluated using the subset of 62 first samples taken. The RF successfully predicted skin hydration levels from the measured microbiome with a MAE of ~ 0.54 on the test set (see Table 2 and Supplementary Table 1). Results from cross-validation (average MAE of ~ 5.70 and standard deviation of ~ 0.46) show that our best model (RF) is stable and performs well on different sets of unseen data, when predicting the corneometer measurements.

The explanations for the corneometer predictions made by our best optimized model (RF) were provided in terms of impactful genera as computed by SHAP. Figure 2a shows a summary dot plot that offers a global overview of the local explanations based on the training data. Figure 2, in fact, explains what the trained model has learned from the training data, e.g., how each genus is impacting the predictions of the phenotypes. The genera are ranked based on their average absolute SHAP impact value across the samples in the training dataset, for example in Fig. 2a *Lactobacillus* is the most impactful genus for the prediction of the corneometer values. In particular Fig. 2a shows how each genus (each row in the SHAP dot plot) contributes, either positively or negatively, to the prediction of the corneometer measurements for the samples (dots) in the training dataset. A positive SHAP impact of a genus for a sample (a dot on the right side of the x-axis) means that the genus contributes to an increase in the predicted corneometer value for that sample. On the contrary, a negative SHAP impact of a genus for a sample (a dot on the left side of the x-axis) means that the genus contributes to a decrease in the predicted corneometer value for that sample. A dot is colored based on the relative abundance of a genus in the corresponding sample. The red dots are the samples for which a genus is highly abundant, and the blue dots are the samples for which a genus is less abundant. See the  Methods section for a detailed description of the explanaibility algorithm (SHAP) that we applied, and the summary dot plots.

The top 30 ranked genera shown in Fig. 2a have a significant impact in the model’s predictions and the relative abundance of these genera (i.e., the values of some of these features) informatively form separated clusters (red or blue) that discriminate positive or negative impact of a genus on the model prediction i.e., high or low skin hydration respectively. We found that an increase in the relative abundance of 11 genera is responsible for predicting higher skin hydration, while an increase in the relative abundance of 19 genera is responsible for predicting lower skin hydration (see Fig. 2a). For example, enrichment of *Lactobacillus, Brevundimonas, Corynebacterium, Finegoldia, Manihot, Collinsella and Enterobacter* is contributing to the prediction of higher skin hydration. On the other hand, for example, higher abundance of *Abiotrophia, Pantoea, Fusobacterium, Parvimonas* is contributing to the prediction of lower skin hydration. Among the genera most determinant of increased skin hydration were *Lactobacillus, Brevundimonas* and *Corynebacterium*, all of which have previously been reported as being associated with skin hydration or skin health^23, 24, 40, 41^. In contrast, the genera *Abiotrophia*, *Kocuria, Veillonella* and *Erwinia* were associated with decreased skin hydration, which also aligns with previous studies^42–44^. As the insights shown in Fig. 2a can be extended to include the entire set of genera, our streamlined approach offers an opportunity to investigate how each skin microbiome genus impacts skin hydration. Furthermore, when we compared the explanations of the training data and of our unseen test dataset (Fig. 2a and Supplementary Figure 6a) we observed a large degree of overlap (100% overlap in the top 20 ranked genera and 86.7% overlap in the top 30 ranked genera overlap) and consistency in the direction in which the genera are driving the predictions of both the training and the test datasets. Note that in Fig. 2 and Supplementary Figure 6 the top genera are ranked based on their mean SHAP value across the samples. As the training and test datasets included different samples, the order of the top 30 genera in the figures could differ slightly.

To investigate the generalizability of our approach we extended the skin hydration analysis to include a UK cohort composed of 278 samples from 102 subjects and the same set of 186 OTUs as for the Canada study. The aim was to validate the skin hydration model and to investigate how the model generalizes across cohorts, while highlighting differences and similarities in the explainability results. We applied the three ML methods to predict skin hydration from two datasets for the UK cohort; 278 samples blocked by subject and 102 first samples taken from the UK cohort. We found that the subset most predictive of skin hydration was, in this case, the 278 samples blocked by subject. We then compared the two best models respectively for the UK and Canada cohorts (Table 2). We observed that the error increased when training and testing the models on the UK samples blocked by subject, compared to when training and testing using the 62-first samples taken of the Canada cohort, resulting in 8.92 (UK) vs 5.54 (Canada) MAE on the test set. In addition, when using our best model (RF) for the Canada samples (trained on the Canada training data) to predict the 278 unseen UK samples, we obtained a MAE of ~ 11.79. Note that corneometer assumes values in a range between [8.1,78.4] with median ~ 27.3 for the UK cohort, while the corneometer range for the Canada cohort is [2.4, 57.2] with a median of ~ 24.36 (see Table 1 and Supplementary Figure 1 for more detail on the distributions). As such, using these ranges, we computed a 6.6 percentage points increase in MAE when using the best model for the Canada samples to predict UK samples (MAE ~ 11.79) compared to using the best model for Canada samples to predict the unseen Canada samples in the test set (MAE ~ 5.54).

Finally, we applied our EAI approach to explore the commonality and differences between the impactful genera for predicting skin hydration in the best models for the Canada cohort (trained on Canada samples) and the UK cohort (trained on UK samples) separately. Supplementary Figure 7 shows the explanations for the UK skin hydration model. We found common impactful genera in both the UK and Canada models. For example, *Lactobacillus* and *Corynebacterium* are among the top 20 impactful genera driving the prediction of more hydrated skin (see Supplementary Figure 7) both of which have previously been reported as being associated with skin hydration or skin health^23, 24, 40^. *Kocuria* is another example of common impactful genus that is driving the prediction of less hydrated skin for both UK and Canada models. In addition, there are some genera in the UK model, such as *Dermacoccus and Ochrobactrum*, that do not appear in the list of the top impactful genera for the Canada model, and vice versa (see Fig. 2a vs Supplementary Figure 7).

### Explainable age model identifies key microbes predictive of age: Canada cohort

A recent study found that the skin microbiome of the hand and forehead was the most predictive of age when compared to the gut and the oral microbiome, yielding predictions within 4 years of chronological age^42^. As such, we applied our EAI approach to predict age from the different subsets of the Canada cohort (Table 2 and Supplementary Table 1). Our best fine-tuned model (RF) was able to predict the subjects’ age from the skin microbiome samples achieving an average error within 6 years of the chronological age (~ 6.36 MAE) on unseen data from the test dataset. Table 2 shows the average MAEs obtained by our best fine-tuned model on the test and the training datasets. Note that the RF provided the smallest difference in MAE between training and test sets when using the 62 first samples (see Supplementary Table 1 reports to compare the performance of each model).

Analogously to the skin hydration model, we applied the SHAP explainability analysis to understand the link between bacterial communities on the skin and subject’s age. Our EAI approach revealed that higher abundance of 26 genera drives the prediction of younger age, while higher abundance of 4 genera contributes to the prediction of older age (see Fig. 2b and Supplementary Figure 6b). The genera most determinant of younger age, when higher in abundance, included *Alloprevotella*, *Granulicatella, Gemella and Lactobacillus* (Fig. 2b), while the genera *Bacteroides, Pseudomonas*, *Bergeyella* and *Bacillus* were predictive of older age for the samples in the training and test sets (Supplementary Figure 6b). Note that the genera are ordered based on the average of their absolute SHAP values across the samples. As the training and test sets include different samples, the rank of the genera might differ slightly when visualized in the SHAP summary dot plots (Fig. 2b and Supplementary Figure 6b). When we compared the explanations for the top 30 genera between the training dataset and our unseen test dataset (Fig. 2b and Supplementary Figure 6b), we observed a large degree of overlap (93%) in the top 30 ranked genera and consistency in the direction in which these genera are driving the predictions of both the training and test datasets.

The model’s explanations indicated that *Propionibacterium* is a key genus for predicting younger age when enriched in the samples of the training and test datasets (Fig. 2b and Supplementary Figure 6b). This is consistent with previous findings that showed a significant decrease in the relative abundance of Propionibacterium in the forehead, cheek and forearm of older subject^45^. This might be related with a decrease in sebum secretion level in older skin^46^. Similarly, *Granulicatella*, driving the prediction of younger age, has been found to be negatively correlated with age^47^, while *Lactobacillus* was linked to anti-aging^48^. *Bacillus*, on the other hand, was found to be dominant in the cheek microbiome of older people^49^; that is in line with the fact that in our model *Bacillus* is driving the prediction of older age when enriched in the microbiome samples.

Finally, we found some overlap between the genera identified as being important for both the skin hydration and age models (30%). However, the majority (70%) of the genera among the top 30 most impactful ones were different (Fig. 2a vs 2b). This enabled the disambiguation of microbes whose effects mainly relate to either skin aging or hydration. In fact, perhaps contrary to expected, these results showed that skin hydration and age are not inexorably linked. This can be observed in Fig. 1b, which shows a very weak link between age and corneometer score (Spearman’s correlation of ~ -0.11).

### Leg skin microbiome accurately predicts menopausal status: Canada cohort

To examine changes in the skin microbiome that are associated with the menopause and to investigate how these changes are also related to aging, we applied our EAI approach to classify samples from the Canada cohort into pre-menopausal and post-menopausal status. The dataset included 324 samples of 18 subjects in pre-menopausal status and 876 samples of 44 subjects in post-menopausal status. We applied the EAI approach to both the 1200 time series samples blocked by subject, to the first samples taken from the 62 different subjects and to the last samples taken from the 62 subjects. We found that using the 62 first samples provided the best predictive performance (Table 3 and Supplementary Table 2). The best fine-tuned model (LightGBM) was able to predict both classes and showed robustness when cross validated, with an F1-score of 0.92 on the unseen samples of the test set. Table 3 shows the F1-score, precision and recall per class computed on the test dataset, as well as the weighted average of the F1-score both on the training and the test data. Supplementary Figure 8a displays the confusion matrix computed on the test dataset, which further demonstrates how LightGBM was able to accurately predict each class. Note that only one sample with the status post-menopausal was wrongly classified as pre-menopausal, while all the samples in the minority class (pre-menopausal) were correctly classified.

We generated insights for the menopausal model by explaining the mechanisms underlying the predictions of our best model (LightGBM) using the best subset (62 first samples taken) from the Canada cohort. Figure 2c shows the explanations for the training dataset and Supplementary Figure 6c shows the explanations for the test dataset, i.e., the impact of the top 25 impactful genera as generate by SHAP.

Since, as expected, menopause and age are correlated, we investigated similarities and differences between the two models taking into account possible confounding factors when predicting age or menopausal status. The menopause model considered a number of genera to be important (e.g., *Lactobacillus*, *Dermacoccus, Granulicatella, Pseudomonas, Bergeyella and Bacillus*) that were already seen as key indicators in the age model, along with a key indicator of skin hydration (*Lactobacillus*). This is perhaps not surprising as menopause usually occurs in middle age and the skin is significantly affected by the aging process and menopause^50^. Interestingly, we noted that *Granulicatella, Dermacoccus* and *Lactobacillus* were amongst the most important genera in the age model that predicted a younger age, and in line with this they also predicted a pre-menopausal status when higher in abundance.

Genera such as *Brachybacterium, Cutibacterium (formerly Propionibacterium), Atopobium, Bacteroides, Porphyromonas, Tannerella, Alloprevotella, Gemella, Eremococcus, Enterococcus, Solobacterium, Dialister, Veillonella, Brevundimonas, Campylobacter and Aeromonas* appear among the most impactful for predicting age, while they do not appear in the menopausal model. This aligns with expectations from previous work, where *Bacteroides, Porphyromonas, and Veillonella* have been directly linked to age via the skin microbiome^49^ and where the expansion of some species of *Cutibacterium, formerly Propiniobacterium,* is known to be favored by younger skin^45^. On the other hand, *Brevibacterium, Blastococcus, Parabacteroides, Alistipes, Streptococcus, Finegoldia, Alphaproteobacteria, Ochrobactrum, Paracoccus* are considered impactful for predicting menopausal status alone and do not appear in the ranked list of most impactful genera of the age model. Many of these genera can be supported by previous work, e.g., with *Parabacteroides* and *Alistipes* abundance being previously linked to menopausal status via the gut microbiome^47, 51^. Additionally, the enrichment of *Streptococcus and Bacillus* in the vaginal microbiome of post-menopausal women has been reported^52, 53^, which supports our EAI approach linkage of increased abundance with the classification of post-menopausal women. The same previous work found the depletion of *Lactobacillus* as the most dramatic change in the post-menopausal microbiome (compared to pre-menopausal) which matches the directionality and impact of this genus here since it is our most impactful feature. Finally, when we compare the explanations for the menopausal model between the training data and our unseen test dataset (Fig. 2c vs Supplementary Figure 6c) we observed a large degree of overlap (96% in the top 25 ranked genera) and consistency in the direction in which the genera are driving the predictions of both the training and the test dataset.

To investigate the relationship between the continuous variable age and the binary variable menopausal status, we computed their point-biserial correlation, which is 0.71 (Fig. 1b). As such, to take into account possible confounding factors, we trained our ML models for predicting age separately for pre-menopausal and post-menopausal sample groups. We found that it was possible to accurately predict age for the two sub-groups, showing that age prediction is robust (see Supplementary Table 3). More precisely, we obtained an error of ~ 5 years on the test set of post-menopausal samples, and an error of ~ 6 years on the test set of the smaller pre-menopausal group. Supplementary Figure 9 shows a comparison of the top 25 impactful genera, as generated by SHAP using the best selected model (RF), between pre-menopausal and post-menopausal groups. Above, we predicted menopausal status from all subjects and identified key genera that were menopause-specific and not highly impactful in the age model. In fact, the overlap between the top 20 most impactful genera between the menopause and the age models was only 40%, demonstrating that the age and menopause models differ from each other and may offer different and unique insights. Here, if we compare our two new age models that separate pre and post-menopausal subjects, this overlap of insights decreases further. For pre- and post-menopausal age models, respectively, we found; an overlap of only 10% between their most impactful genera, an overlap of 25%/30% compared to the previous age model and an overlap of only 15%/15% with the menopausal model. This indicates that when we separate pre- and post-menopausal groups to predict age, the insights differ not only significantly from those generated when the groups are combined to predict age, but also more significantly from those of the menopausal predictive model. Therefore, the stratification of subjects may offer additional insights that are more specific to phenotypic values. The most impactful unique genera for age prediction in the pre-menopausal group (absent from all other models) included *Rothia, Fusobacterium and Prevotella*, which have been previously linked to age with the same positive correlation we observed here through explainability^45^. The most impactful unique genera for age prediction in the post-menopausal group (absent from all other models) included *Pasteurella, Mobiluncus and Deinococcus*.

### Key microbes on the leg discriminate smokers accurately: Canada cohort

With the aim of investigating skin microbiome variations associated with smoking habits, we applied our EAI approach to predict smoker vs non-smoker subjects from the leg skin microbiome of the Canada cohort. We applied the EAI approach to both the 1200 time series samples blocked by subject, to the first samples taken from the 62 different subjects and to the last samples taken from the 62 subjects. The dataset included 286 samples from 19 smokers and 914 samples from 43 non-smokers. We found that using the 62 first samples provided the best predictive performance (Table 3 and Supplementary Table 2). The best fine-tuned model (XGBoost) was able to predict both classes with an F1-score of 0.85 on the unseen samples of the test set. Table 3 shows the F1-score, precision and recall per class computed on the test dataset, as well as the weighted average of the F1-score both on the training and the test data. Supplementary Figure 8b displays the confusion matrix computed on the test dataset, which further demonstrates how XGBoost was able to accurately predict each class.

We found that the prediction of smokers is driven by higher abundance of 11 genera (including *Brevibacterium, Gemella, Bergeyella, Stenotrophomonas, Lactobacillus, Campylobacter, Rothia, Acinetobacter and Actinomycelates*), while 9 genera (including *Faecalibacterium, Paracoccus, Brachybacterium, Pantoea, Capnocytophaga, Bacteriodes, Rhodococcus and Turicella*) were the most impactful for predicting the class non-smoker when higher in abudance (Fig. 2d and Supplementary Figure 6d). When we compare the explanations between the training data and our unseen test dataset (Fig. 2d vs Supplementary Figure 6d) we observed a large degree of overlap (100% in the top 25 ranked genera) and consistency in the direction in which the genera are driving the predictions of both the training and the test datasets.

Finally, we examined the split of smokers vs non-smokers against age. The age of smokers varies from 21 to 65 years old, while the age of non-smokers varies from 40 to 64 years old. To better quantify the relationship between age and smoking status we trained a Logistic Regression model to predict the likelihood of smokers and non-smokers from chronological age. Supplementary Table 4 reports precision recall and F1-score per class computed on the unseen samples in the test dataset. The results show that the trained classifier is not accurate as it is not able to correctly predict samples in the smaller class, e.g., smokers. Furthermore, we computed the point-biserial correlation between chronological age and smoking status, which consistently gave a low correlation of ~ 0.14 (Fig. 1b).

## Discussion

To summarize the explainability results across phenotypes, we compiled Table 4 and Supplementary Table 5 illustrating both the overlapping and uniquely impactful skin microbes (in Fig. 2) for the phenotype prediction of the 62 first samples taken from Canada cohort. Our study indicated that some members of the microbiome play a general role in wellbeing, as they are involved in consistently predicting more than one of the phenotypes (skin hydration, age, smoking and/or menopausal status) when they are enriched in the samples (see agreement in the top half of Table 4). The genera in Table 4 are impactful for predicting at least two phenotypes each. The genus at the top, *Lactobacillus,* is impactful for predicting higher skin hydration, younger age and pre-menopausal status when higher in abundance. On the other hand, higher abundance of *Bergeyella* is impactful for predicting lower skin hydration, older age, post-menopausal status and smoking status. Nevertheless, accurate predictions of each phenotype require distinct combinations of impactful genera (see Supplementary Table 5, Fig. 2 and Supplementary Figure 6). For example, higher abundance of *Abiotrophia* and *Kocuria* is uniquely driving the prediction of lower skin hydration, while *Atopobium* and *Enterococcus* are most impactful only in the age prediction model. Higher abundance of *Alistipes* uniquely drives the prediction of post-menopausal status, while higher abundance of *Faecalibacterium* identifies non-smokers (Supplementary Table 5). *Pseudomanas* is an example of a genus having opposite impacts on two phenotypes (older age and pre-menopausal status). However, in the menopausal model the impact of *Pseudomonas* is close to zero (as we can see in Fig. 2c the dots are close to zero on x-axis) meaning that *Pseudomonas* is not the most discriminating feature for menopausal status.

**Table 4:**
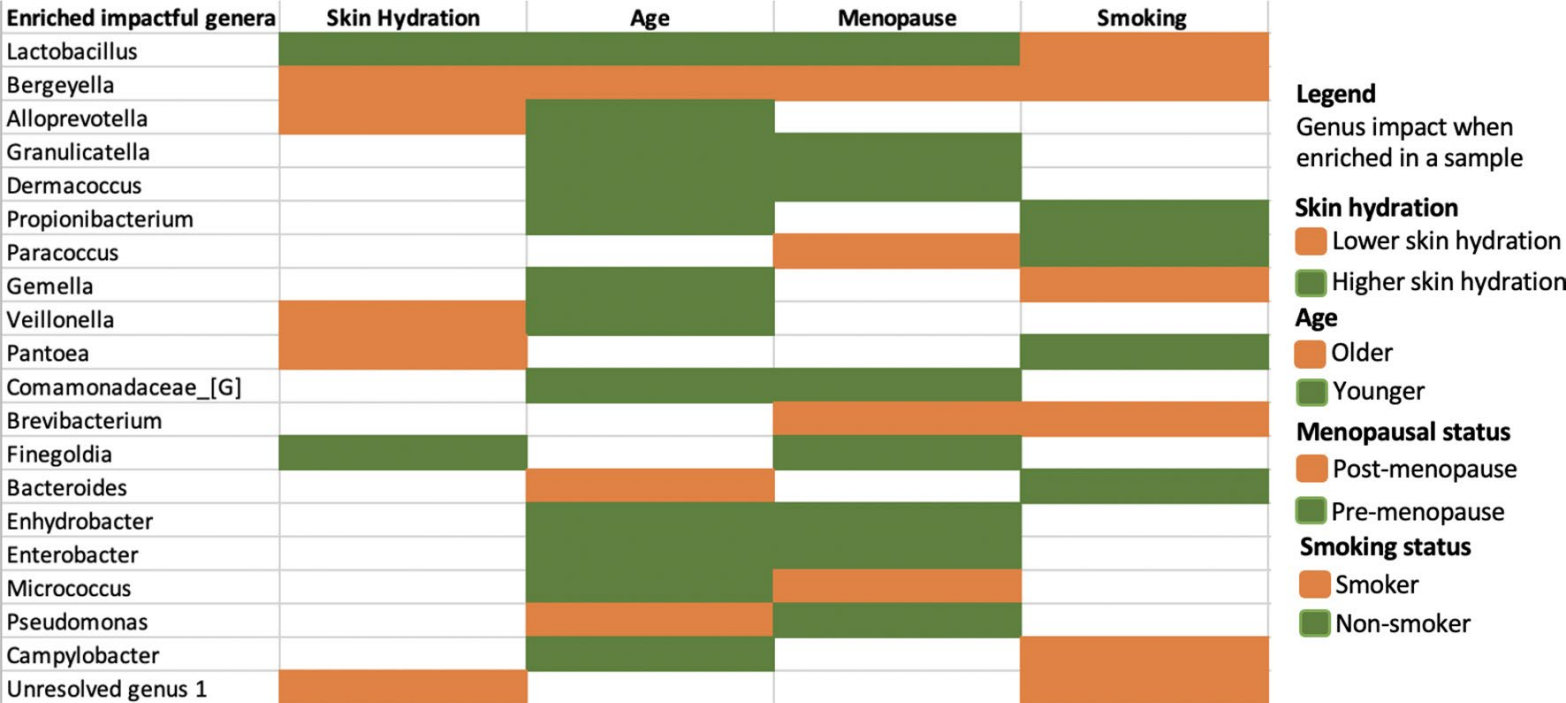
Summary of insights inferred from our EAI framework when predicting skin hydration, age, menopausal status, and smoking status for the 62 first samples taken from the Canada cohort.

The table shows the union of the most impactful genera for each phenotype. The colors (green or orange) reflect the impact that each genus has in predicting a particular phenotype when higher in abundance. For example, higher abundance of Lactobacillus contributes to the prediction of a higher skin hydration (green), while higher abundance of *Bergeyella* drive the prediction of lower skin hydration (orange). The legend on the right shows the color code for each phenotype values.

**Figure 3:**
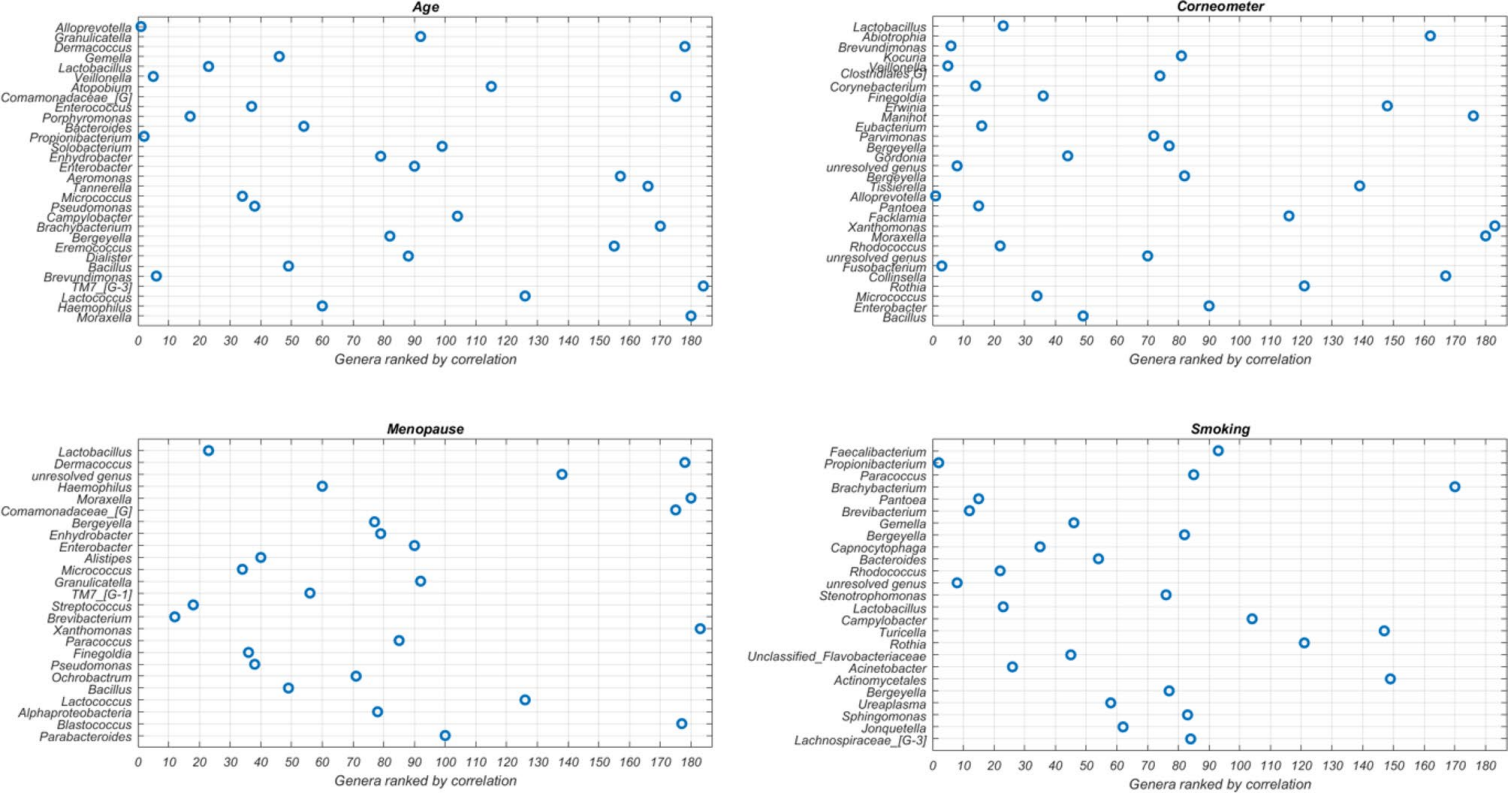
Feature ranking from statistical test. Visualization of the most impactful genera per phenotype. On the y-axis the ranked impactful genera as generated by SHAP (the most impactful genus at the top) are shown. On the y-axis the rankings in independent statistical tests (the most significant (smallest p-value) genus is on the left) are shown. Note that the total number of genera is 186. This image has been generated using Matlab R2017a at https://www.mathworks.com/products/matlab.html.

When we compared the impactful genera from our EAI approach to the results from statistical tests that independently associate each genus with the phenotype (Fig. 3), we observed overlap between the top genera from our EAI approach and those highly ranked according to statistical testing, however, there were notable exceptions also. The 2nd most impactful genus for predicting skin hydration, *Abiotrophia*, was ranked 90th, while our EAI approach sensibly links its high abundance with low skin hydration. This latter result is in agreement with previous studies that found *Abiotrophia* positively associated with skin infections such as Psoriasis^42^. *Dermacoccus*, *Atopobium and Comamonadaceae* are among the top eight impactful genera in the age model but only ranked over 100 from statistical test. Strikingly, *Dermacoccus*, a genus of *Moraxellaceae, Moraxella, and Comamonadaceae* are among the six most impactful genera in the menopausal status model but are all ranked significantly lower (over 100) using standard microbiome analysis. For smoking, the top ML feature *Faecalibacterium* was only at rank 93 according to the statistical test and other five genera were ranked over 100. This indicates the set of most predictive genera inferred by our EAI approach can differ from the ones highlighted by a standard statistical approach that considers each genus independently. As such, our approach can be used to complement standard statistical methodologies to investigate predictive microbial features, at any taxonomic level, whose combined contribution to the phenotype could be missed otherwise.

Our findings on the predictive power of the leg skin microbiome point to the possibility of using more accessible microbiome samples to investigate phenotypes (e.g., smoking status) that are not believed to be directly associated with the microbiome of the sampled body site (e.g., skin microbiome of the leg). The lower abundance of *Faecalibacterium* observed previously in the gut microbiome of smokers^54^ is in agreement with the fact that we found low abundance of *Faecalibacterium* on the leg to be the most impactful for predicting the class of smoker. Additionally, higher levels of *Brachybacterium* in the lower respiratory tract microbiome were previously found in non-smokers^55^, and similarly higher abundance of *Brachybacterium* was found predictive of non-smokers through explainability. Finally, *Brevibacterium* was identified in the bacterial microbiota of a large range of commercial cigarette products^56^, as such, this supports our linkage of higher abundance of *Brevibacterium* to the prediction of smokers. The similarity between the genera that discriminate smokers from non-smokers in our skin model and the genera identified as discriminators of smokers in previous studies of the oral and gut microbiome is encouraging. Despite the fact that smoke is not in direct contact with skin on the leg, we found that the leg skin microbiome likely changes in response to smoking, and in a manner that is consistent with previous findings from different body sites. This further confirms systematic changes occurring across the human body in cigarette smokers. It also suggests the microbiome of distant body sites may be affected by smoking, hence pointing to the fact that extremely complex interactions between body locations or tissues are yet to be understood.

Likewise, microbiome sequencing from vaginal swab samples found a similar membership of major vaginal bacteria at the genus level in pre- and post-menopausal women but with altered proportions. A previous study observed the most abundant genus in the vaginal microbiome of pre-menopausal women to be *Lactobacillus* (64.4%), and its levels were found to be much lower in post-menopause (24.4%), replaced by *Streptococcus* (5.1%) among others^52^. This reflects our observations where *Lactobacillus* is the most impactful feature and when it is enriched, it predicts premenopausal status, while *Streptococcus* is predictive of post-menopausal status when more abundant (see Fig. 2c). Here the skin microbiome appears to share similarities with the vaginal microbiome which could explain its ability to predict menopausal status. Furthermore, additional impactful genera from the menopause model can be supported by previous work in the gut microbiome, e.g., with *Parabacteroides* and *Alistipes* abundance being previously linked to menopausal status^47, 51^.

We took into account possible confounding factors when predicting the four phenotypes for the Canada study. Of the four phenotypes, only age and menopause were found to be correlated (~ 0.71 point-biserial correlation, Fig. 1b). To address this, we showed that we were able to accurately predict age separately for the pre- and post-menopausal groups, demonstrating that age prediction is robust despite possible confounding factors like menopausal status. We then compared the age model explanations among those groups. Through our explainability analysis we demonstrated how the age and menopausal models share common impactful genera, while still considerably differing from each other. For instance, unique genera to the age model were *Bacteroides, Porphyromonas, and Veillonella* that have been directly linked to age via the skin microbiome^49^ and *Cutibacterium, formerly Propiniobacterium,* that is known to be favored by younger skin^45^. Another example of impactful genera unique to the age prediction model specifically in the pre-menopausal group included *Rothia, Fusobacterium and Prevotella* (Supplementary Figure 9), which have been previously linked to age^45^. Therefore, although the phenotypes are correlated, the age and menopausal models can offer insights that are phenotype specific.

We also showed that some of the insights on skin hydration, inferred through explainability, are consistent with previous findings. For example, the genera most associated with increased skin hydration were *Lactobacillus, Brevundimonas* and *Corynebacterium*, all of which have previously being associated with skin hydration or skin health^23, 24, 40, 41^. Moreover, two of these genera, *Lactobacillus* and *Corynebacterium* are among the most impactful genera in both the Canada and UK skin hydration models.

We observed commonalities and differences in the impactful genera of the UK and Canada skin hydration models. Differences in impactful genera and predictive performances between the two models might be due to: a) the different distributions of corneometer values and age between the Canada and UK cohorts, b) the disparity in dimensionality of the two datasets, c) country specific differences in microbiome composition, and d) the different depths of coverages and/or represented microbes between the two datasets.

Finally, while predictive performance is important, the focus of this work is the explainability of the ML models.

Understanding why a model makes certain predictions can be as important as the predictions’ accuracy. However, the “black box” nature of the most accurate ML models, often sophisticated, makes even experts struggle to interpret them. This can create an undesirable compromise between complex high accuracy models and interpretability. Explainability methods can open the “black box” and consequently help build trust in the model’s predictions. However, the model explanations should be treated with a level of doubt commensurate with the inaccuracy of the model’s predictions.

Providing model explanations can help us to understand the link between microbiome composition and phenotypes, and also importantly build trust in the model predictions. As the insights shown in Fig. 2 and Supplementary Figure 6 can be extended to include the entire set of genera, our streamlined approach offers an opportunity to infer new insights by investigating how each skin microbe impacts the phenotype prediction. We propose that all genera with a relatively high impact in the predictive models are likely to either play a biologically causative role or offer value as a non-causative indicator organism and thus are worthy of additional investigation.

In this study we investigated microbial taxa at genus level, however our EAI approach can be similarly applied to the investigation of changes at species or strain level. Moreover, while we performed a particular analysis with one bioinformatics pipeline (to identify genera and abundances from bacterial 16S rRNA gene sequencing), this approach extends to exploring how microbial features from shotgun metagenomic sequencing are impactful in predicting phenotypes. This would involve taking into account the entire genetic material of a microbial community (including fungi and viruses), providing insights related to genes and their associated biological functions. In general, the ability to predict specific phenotypes from non-invasive microbiome sampling can perform a similar revolutionary role as the collection and sequencing of human DNA from cheek swabs, which has powered the gathering of massive data sets and thus the genomic revolution in personalized medicine.

## Conclusion

We developed an explainable AI (EAI) approach to predict phenotypes and explain the predictions in terms of changes in microbiome composition related to diverging phenotypic values. The results presented in this study demonstrate the power of explainable AI in accurately predicting diverse phenotypes such as skin hydration, age and, surprisingly, menopausal and smoking status from the leg skin microbiome, and in inferring microbial signatures associated with each phenotype. In addition, we investigated the application of the skin hydration model to a second, independent cohort to compare the predictive performance and explainability of the model.

Our study focused on personalized care and wellbeing. However, it is straightforward to appreciate how this approach is broadly applicable in healthcare, representing an advancement in the deployment of microbiome-based and non-invasive diagnostics for practitioners (e.g., dermatologists) and in the design of personalized treatments. Impactful future work will likely involve additional independent studies and analysis at species or strain level to investigate health-related phenotypes. Moreover, future work will involve the extension of our EAI approach to incorporate multiple explainability methods as well as transfer learning methods with the aim of improving the generalisability of ML models and their explanations across different cohorts.

Our EAI approach will enable the community to expand and build on this initial work as the explanations may offer new insights into the complex interactions between microbes and their host, possibly leading to new interventions to adjust the microbiome for improved health outcomes.

## Methods

**Figure 4:**
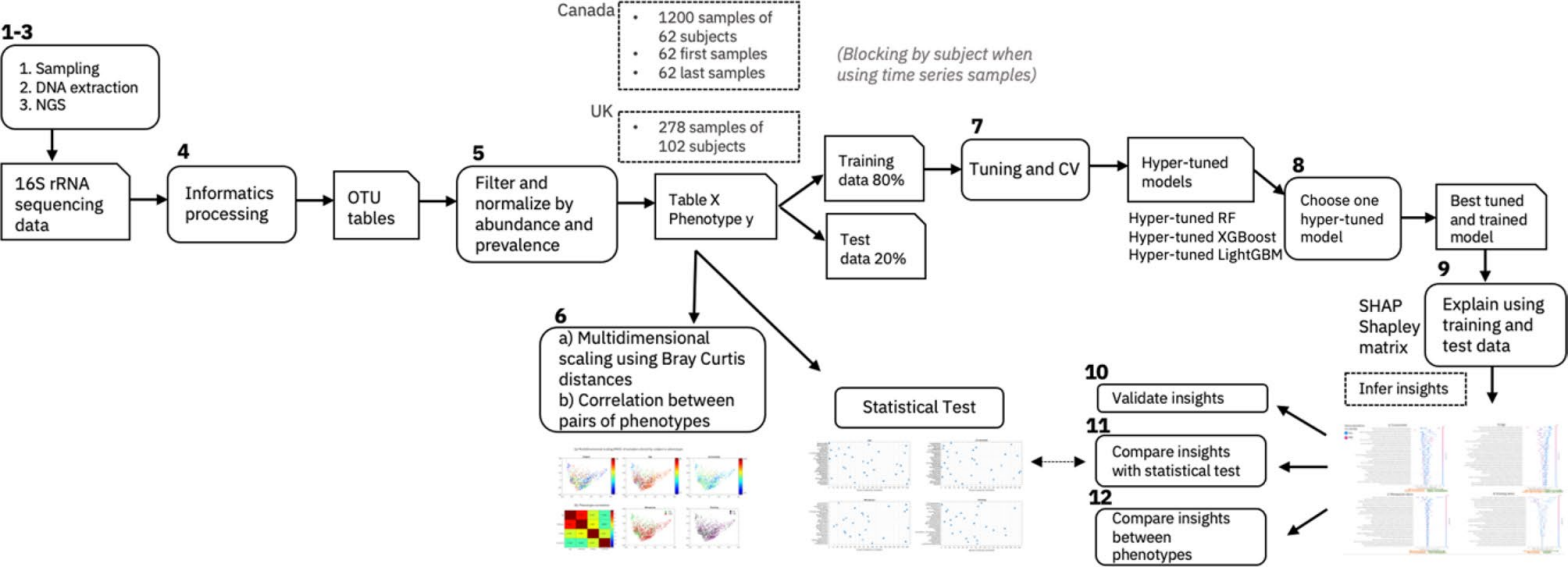
Explainable AI (EAI) approach for microbiome data. This figure shows the main steps (1–12) of our workflow. The main steps include microbiome sampling and 16S rRNA sequencing (1–3), bioinformatic pre-processing (4), filtering of the resulting OTUs tables (5), multi-dimensional scaling of pairwise Bray–Curtis dissimilarities between samples performed on different subsets of the Canada cohort, and phenotype correlations (6); hyper-parameter tuning and cross validation of each ML model (RF, XGBoost and LightGBM) using the training dataset to predict the host phenotype from different subsets of the Canada and UK cohorts—blocking by subject when using time series samples (7); training each of the fine-tuned ML models (hyper-tuned RF, XGBoost and LightGBM) on the training data and testing it on the test data. To provide explanations, choose the best performing hyper-tuned and trained model (e.g., tuned LightGBM) for each cohort (8); explaining the predictions of the chosen model using the training and the test data (9), validating the insights inferred from the model explanations against the literature (10), performing statistical testing for feature ranking and finally comparing the insights from the statistical test to the insights inferred by using the explainability algorithm method (SHAP) (11), comparing the inferred insights on the impactful genera across different phenotypes (12).

The first steps of our EAI approach, shown in Fig. 4, consist of microbiome sampling, sequencing, informatic pre-processing and filtering (e.g., steps (1–5) detailed in the following sections) to obtain the closed reference OTU tables for both the Canada and the UK cohorts. Before proceeding with phenotype prediction, we projected multidimensional scaling of pairwise Bray–Curtis distances between samples to check if the samples were separable by subject or any of the phenotype (step 6) and computed correlations between each pair of phenotypes to investigate possible confounding factors. Next, we randomly split the dataset in two parts, 80% for training and 20% for testing. The ML models (RF, XGBoost, LightGBM) were tuned on the training dataset to perform the specific prediction task: regression for predicting skin hydration and age; classification for predicting menopausal and smoking status (step 7). Once the best hyper-parameters were selected, each tuned model was trained and tested. We then compared the predictive performances of the models for different prediction tasks and different subsets of samples and selected the best model for each combination (step 8). Finally, we generated explanations of the predictions for the best models that were compared to the insights derived from the statistical test analysis (steps 9–12). In the following subsections we discuss in detail each step of our workflow.

### Clinical design and data collection

#### Study participants

A total of 63 Canadian women between 21–65 years of age (average 52.71 years) took part in this double blind randomized, balanced application study, between April and July 2017. In addition, 102 women aged between 19–55 years (average 38.03 years) took part in a UK based study. Key inclusion criteria included subjects being in good general health, not being pregnant or lactating and having intact non-diseased skin with minimal hair (on test sites). Key exclusion criteria included pregnancy or lactating mothers and use of moisturisers on the proposed test site 3 or more times per week. A full list of the inclusion and exclusion criteria can be found in Supplementary Notes.

#### Study design

The study was carried out by an independent third party. Study visits included visual and instrumental assessments, as well as buffer scrub sampling. Additionally, subjects were asked to confirm their age, smoking and menopausal status at the start of the study. There was no conditioning (wash-out) phase required for this study.

#### Assessment of skin condition

Visual assessments of leg skin include dryness and erythema grading. Visual dryness assessments were made by an expert assessor with dryness scored on comparable scales from 0–6 (Canada) and 0–4 (UK) both with ½ point increments allowed. The descriptors for 0–4 skin condition in terms of severity are similar across scales. Instrumental assessments (Skicon, Corneometer, and pH) were taken following the visual assessments and a total combined acclimation period of a minimum 25 min to an environmental controlled room. The Skicon-200EX (IBS Co./AcaDerm) measures skin hydration by skin surface conductance, and for the Canada study a Corneometer CM 825 Courage + Khazaka) was used to measures skin hydration by capacitance (the skin’s ability to conduct a small electrical current). For logistical reasons the UK study used a Courage and Khazaka Multi-Probe Adaptor Corneometer (MPA 6) for the same purpose, both instruments are widely used for this purpose and offer comparable function, performance and sampling area. Skin surface pH in the Canada study was measured using a pH meter (Courage + Khazaka).

The corneometer measurement was the most amenable for machine learning models (see Phenotype analysis in Supplementary Notes), therefore we focused on it in this study. Between 3 and 5 corneometer readings per test site were obtained and the probe was moved slightly (overlapping may occur) with each reading, but still within the site. Pressure on the skin surface was measured by means of a probe spring and was 3.5 Newtons onto the area measured. The area of skin in contact with the probe was 49 mm^2^.

#### Microbiome sampling

After all instrumental measures, cup scrubs buffer washes were collected using the method of Williamson and Kligman^57^. Using a sterile plastic disposable pipette 2.5 ml of buffer wash solution (PBS + 0.1% Triton X-100) was aliquoted into the sampling ring and gently agitated for one minute with a sterile rod. The sampling fluid was collected using a sterile disposable pipette and placed into a sterile tube. The sampling procedure was repeated with another 2.5 ml aliquot of buffer wash material. After agitation, this aliquot was added to the first. Samples were frozen at -20 ºC within 10 min until DNA extraction. Skin microbiome samples were taken by the cup scrub method at four different points, with either one or two samples being taken on each leg, as described above. Samples were taken from the same individual at different time points giving an overall total of 1234 microbial samples from the 63 subjects. Note that the only subject (with 34 time series samples) in peri-menopausal status was excluded from our analysis.

#### DNA extraction

DNA extractions were carried out by QIAGEN (Germany). Frozen buffer scrub samples were allowed to thaw at room temperature and cell pellets sedimented by centrifugation at 13,000 rpm, 4 °C for 10 min. Following removal of the supernatant the cell pellet was resuspended in 500 µl TE buffer and transferred to a 96-well Lysing Matrix Plate B (MP Biomedicals, USA). Addition of 3 µl of Ready-Lyse lysozyme (Epicentre, 250U/µl) was followed by incubation with agitation at 300 rpm, 37 °C for 18 h. Following lysis DNA was extracted and purified using the QIAmp UCP DNA Micro Kit (Qiagen, Germany) following the manufacturer’s instructions. DNA was eluted into AUE buffer and frozen until processing for sequencing library preparation.

#### NGS library preparation and sequencing

Oligonucleotide primers targeting the V1-V2 hypervariable region of the 16S rRNA gene were selected. PCR primers (details in Supplementary Information) were a modified version of the standard 28F and 338R primers which contain additional recognition sequences to facilitate nested PCR to add Illumina sequencing adapters and index sequences to resulting amplicons. PCRs consisted of 0.25 μl (10 μM) of each primer, 10 μl of HotStar Taq Plus Mastermix (Qiagen), 5 μl of template DNA and 4.5 μl molecular grade water (Ambion, Thermofisher). Samples were amplified using the following parameters: 95 °C for 5 min, then 10 cycles of: 94 °C for 45 s, 65 °C for 30 s, and 72 °C for 60 s, with a final extension of 10 min at 72 °C using a Dyad Thermocycler (MJ Research). PCR products were purified using Ampure SPRI Beads (Beckman Coulter, California, USA). A second round PCR incorporated Illumina adapters containing indexes (i5 and i7) for sample identification utilising eight forward primers and twelve reverse primers each of which contained a separate barcode allowing up to 96 different combinations. Second round PCRs consisted of 0.5 μl (10 μM) of each primer, 10 μl of 2 × Kapa Mastermix (Roche, Switzerland) and 9 μl of purified sample from the first PCR reaction. Samples were amplified using the following parameters: 98 °C for 2 min, then 15 cycles of; 20 s at 95 °C, 15 s at 65 °C, 30 s at 70 °C with a final extension of 5 min at 72 °C. Samples were purified using AMPure SPRI Beads before being quantified using Qubit fluorimeter (Invitrogen, California, USA) and assessed using the Fragment Analyzer (Advanced Analytical Technologies, Iowa, USA). Resulting amplicon libraries were taken forward and pooled in equimolar amounts using the Qubit and Fragment Analyzer data and size selected on the Pippin prep (Sage Science, Massachusetts, USA) using a size range of 300–700 bps. The quantity and quality of each pool was assessed by Bioanalyzer (Agilent Technologies, California, USA) and subsequently by qPCR using the Illumina Library Quantification Kit (Kapa) on a Light Cycler LC480II according to manufacturer’s instructions (Roche, Switzerland). Each pool of libraries was sequenced on a flowcell of an Illumina HiSeq with 2 × 300 bp paired-end sequencing using v3 chemistry (Illumina, California, USA).

### Informatics processing

The sequence data was processed as follows. Illumina adapters and PCR primers used for initial 16S rRNA gene amplification were removed from each fragment using Cutadapt version 1.14^58^. Sickle version 1.33^59^ was used to quality trim DNA reads using a minimum quality value of 28. Reads shorter than 100 bp following quality trimming were discarded. If a read was discarded during this process its corresponding pair was also discarded. Considering our usage of 16S rRNA sequencing data, reads passing filtering were merged using PANDAseq^60^ version 2.9 to generate overlapping 16S rRNA marker gene contigs for each sample, with a minimum overlap of 20 bp and a minimum amplicon length of 200 bp. The resulting overlapped reads were de-replicated using VSEARCH version v1.9.6^55^ and searched against a BLAST database composed of the HOMD, HOMD extended and Greengenes sequences (HOMDEXTGG) described by Al-Hebshi et al.^61^. Genus level taxonomic classification was then performed for both datasets in a closed reference fashion against the same database HOMDEXTGG version 14.51, as previously described in Ref.^61^, at 99% identity across 98% of the read length. Reads not classified by this process were discarded. Reads were marked *unresolved* when there was no agreement between the databases they were compared against. This process resulted in 1260 taxonomically classified operational taxonomic units (OTUs). The resulting classification table and associated representative sequences, selected as the most abundant sequence for each classified taxon, were used as inputs for QIIME^62^ (Quantitate Insights into Microbial Ecology) version 1.9.1.

The microbial sequence count table and metadata, in biom file format, was loaded into the calour library^63^. The loading process filtered out samples with fewer than 1000 reads and then rescaled each sample to have its counts sum up to 1000 (by dividing each feature frequency by the total number of reads in the sample and multiplying by 1000). After loading, the data underwent two rounds of filtering and the remaining features were collapsed at the genus level. For these rounds of pre-processing filtering, an open-source python library called calour http://biocore.github.io/calour/ was used. The first round of filtering removed low-abundance features, e.g., OTUs with total count less than 10 across all samples (calour.experiment.filter_abundance(10)). The second filter removed OTUs with low prevalence, e.g., features occurring in < 1% of the samples (calour.experiment.filter_prevalence(0.01)). Three bacterial genera *(Burkholderia, Dietzia, Mycobacterium)* were removed from the analysis as they had been identified as contaminants of soil and water systems therefore not characteristic of the skin microbiome. After filtering and collapsing the OTUs at genus level we obtained a total of 186 OTUs (genera) and 1200 samples of 62 subjects of the Canada cohort (after excluding a Canadian subject with 34 samples due to being the only one in perimenopause) and 278 samples of the UK cohort that were used in subsequent analyses). As a final step, the genus counts were rescaled to be fractions of the total count per sample, so that the sum of each sample’s genus counts is 1.

#### Phenotype correlations and sample visualization

Pairwise phenotype correlations were computed by Spearman’s correlation (Matlab function corr) for all other comparisons, except for binary vs. continuous phenotypes point-biserial correlation was used (Fig. 1b). The *function Point biserial* from the Matlab file exchange was used. Each subject only contributed once for each correlation measure. For corneometer the median of measured values per subject was used. To compute the correlation for binary phenotypes (smoking and menopausal status) we used the encoding smoking no/yes = 0/1, menopause pre/post = 0/1. Pairwise sample distances were computed with Bray–Curtis distance between feature (genus) vectors per sample (function f_dis from the *Fathom toolbox* for Matlab). Multidimensional scaling (Matlab function cmdscale) was applied to project the distances into two-dimensional sample representations for visualization (Fig. 1a).

#### Feature ranking by statistical testing

Statistical tests were applied to rank features as follows. For the binary phenotypes (smoking and menopause status), two-sample t-test (Matlab function ttest2) was applied independently to each feature, and then features were ordered by p-value from most to least significant (Fig. 3). For age and corneometer, Spearman’s correlation (Matlab function corr) between each feature and phenotype was applied, and features were ordered by absolute value of the correlation. When there were multiple measures of corneometer per subject, the median value was used.

### Machine learning models and explainability

#### Tuning, training and cross validating the ML models

For each considered subset of the Canada cohort (62 first samples taken, 62 last samples taken and 1200 time series samples blocked by subject) and of the UK cohort (102 first samples taken and 278 timer series samples) we evaluated the application of three machine learning models—random forest (RF)^17^, XGBoost^18^, and LightGBM^19^- (steps 7–8 in Fig. 4) to predict the host phenotypes (skin hydration, age, menopausal status and smoking status). For the UK cohort we applied the ML models only to predict corneometer measurements. We used the scikit-learn implementation of the RF (RandomForestClassifier and RandomForestRegressor), the python implementation of XGBoost and the python implementation of LightGBM (LGBMClassifier and LGBMRegressor). The first step, before tuning and training our models, was to standardise the data using the scikit-learn implementation of the StandardScaler function. We then split our samples set in the training set (80% of the entire dataset) and the test set (the remaining 20% of the dataset) using the scikit-learn function train_test_split with settings shuffle = True (and stratify = target_variable for classification tasks). When we used time series samples, we blocked our analysis by subject, i.e. we ensured samples of the same subject were not present both in the training and in the test datasets, by using the scikit-learn implementation of GroupShuffleSplit with settings n_split = 1 and test_size = 0.2 and groups = subject_ids.

RF, XGBoost, and LightGBM were hyper-tuned using the training dataset, for the different classification and regression tasks and for the different sample subsets. The hyper-parameters of each ML model were tuned using the training dataset and by performing five-fold cross validation. Hyper-parameter optimization (HPO) of the RF and XGboost consisted of 200 iterations of a random search, where each iteration used a different combination of randomly selected hyper-parameters. We used the scikit-learn implementation of the random search (RandomizedSearchCV). LightGBM was optimised using an HPO framework called Optuna^64^ (optuna — Optuna 2.3.0 documentation). Supplementary Table 6 reports the best hyper-parameters for the best models that were selected (RF, XGBoost and LightGBM).

Once tuned and trained on the training dataset, the optimised models were applied to predict the phenotype of unseen samples of the test dataset. Performances on the test set were compared by looking at the lowest MAE for regression tasks (corneometer and age prediction) and the highest F1-score, precision and recall for classification tasks (smoking and menopausal status prediction). We used scikit-learn implementations of MAE, F1-score, precision and recall. For classification tasks, we also computed the confusion matrix based on the predictions on the test data using the scikit-learn implementation confusion_matrix.

To examine the stability and robustness of our optimized models on different randomly selected unseen datasets (i.e., different test sets) we ran tenfold cross validation (10-CV) for each model and each dataset. For regression tasks we used the scikit-learn implementation of KFold with parameters n_splits = 10 and shuffle = True. For classification tasks we used the scikit-learn implementation of StratifiedKFold with parameters n_split = 10, shuffle = True that preserve the percentage of samples of each class. When we used time series samples, we blocked our analysis by subject, i.e., we ensured samples of the same subject were not present both in the training and in the test data during cross validation. To do so we used the scikit-learn implementation of GroupShuffleSplit with settings n_split = 10, test_size = 0.2 and groups = subject_IDs. Finally, we compared the predictive performances of our models (Tables 2, 3 and Supplementary Tables 1 and 2). For each prediction task, we selected the best ML model, i.e., the model that provided the best score, the least difference in performance scores between the training and the test dataset (i.e., least overfitting), and we examined the best model’s insights. Note that we used a global random seed (set to 42) for our analysis. Supplementary Table 7 shows the python packages used in our anaconda environment.

#### Explaining the ML models with SHapley Additive exPlanations (SHAP)

Examining the ML models for explanations of their predictions is an important active field of research. Various methods have been proposed to help users interpret the predictions of complex models. Work such as DeepLIFT^65^, DeepExplain^66^, for example, inspect the gradients of the models to determine the impact of different features. To investigate the mechanisms underlying the predictions, we used an explainable AI algorithm called SHapley Additive exPlanations (SHAP)^20^ (step 9 of Fig. 4). One of the main advantages of SHAP is its ability to work with any machine learning model: tree-based models, such as XGBoost and LightGBM, as well as kernel-based and deep learning models. In fact, SHAP unifies six different explainability methods^20^. For example, SHAP connects LIME and Shapley values in the Kernel SHAP (or Deep Lift with Shapley values in the Deep Kernel) allowing a better understanding of both methods. SHAP combines game theory with local explanation enabling accurate interpretations of how the model predicts a particular value for a given sample. The explanations are called local explanations as they show how each feature is contributing, either positively or negatively, to the prediction of a particular instance. In addition to providing the ranked list of important features for a ML model, one advantage of SHAP over other feature importance methods is that it also explains how each of these impactful features is contributing (positively or negatively) to the prediction of specific phenotypic values. The local explanations reveal subtle changes and interrelations that are otherwise missed when these differences are averaged out. Another advantage of SHAP is that, unlike other explainability methods (e.g., LIME^67^), it offers a global view of local explanations through the Shapley value matrix that it generates (one row per data instance and one column per feature). Examples of global visualization of the Shapley values are the summary dots plots (Fig. 2 and Supplementary Figure 6) that allow for interpretation of the entire model. The summary dot plots show the impact that each feature has on the prediction of the target variable for instances that share similar feature values (e.g., the clusters of red or blue dots in Fig. 2). As such, they offer insights into how different feature values drive the predictions of diverging phenotype values (e.g., hydrated vs dehydrated skin). By comparing the predictive performances of our ML models, we selected the best model at predicting each phenotype that was then explained using SHAP.

We used the python implementation of SHAP available at (https://github.com/slundberg/shap). To obtain the appropriate SHAP explainer for RF, XGBoost and LightGBM we combined the tree explainer^68^ with the best optimised model by applying the function shap.TreeExplainer(tuned_model). We used the resulting explainer to compute the matrix of Shapley values for both the training and the test datasets (explainer.shap_values(X_train) and explainer.shap_values(X_test)). We then use the SHAP values to produce the summary dot plots (Fig. 2 and Supplementary Figure 6) for both the training and the test datasets using the function shap.summary_plot() with parameter plot_type set to dot or bar.

A *SHAP summary dot plot* provides a visualization of the Shapley values, therefore a global overview of the local explanations for a set of samples. The y-axis is the list top impactful features for a model in descending order, therefore each row is a feature. The dots in each row are the data points, or samples, and are colored by the original feature value, that in this case is the genus abundance. The x-axis is the SHAP value or impact: how important is a feature for a particular sample in the model. A positive SHAP value/impact of a feature for a sample (the dot is on the right side of the x-axis) indicates that the feature (e.g., genus) has a positive impact in predicting the target value (e.g., the phenotypic value), while a negative SHAP impact (the dot is on the left side of the x-axis) indicated that the feature has a negative impact on the prediction. For each row (feature) the red and blue dots can form separate clusters that are positioned towards the right or left side of the x-axis. This indicates that the overall the feature (e.g., genus) tend to have a similar impact (positive or negative) for samples in which it has similar feature values (e.g., higher abundance).

### Ethical approval and consent to participate

Written informed consent was obtained from all enrolled individuals. The study protocol was reviewed and approved by Chesapeake IRB, an independent ethics committee. Unilever consults independent ethics boards for study protocol approval to ensure a high quality and unbiased assessment. The methods and protocol were carried out in accordance with the approved ICH/GCP guidelines.

### Consent for publication

All authors have consented to this publication.

## Supplementary Information

Supplementary Information 1.

Supplementary Information 2.

## Data Availability

The sequencing data used in this study have been deposited in the National Center for Biotechnology Information (NCBI) Sequence Read Archive (SRA) with ID PRJNA61366. The data will be made publicly available upon publication. Access for review purposes only is currently available at https://dataview.ncbi.nlm.nih.gov/object/PRJNA613666?reviewer=dill1h0vmlfl3h7f8iaesfh1br. The OTU tables have been uploaded as supplementary file and will be made publicly available upon publication. The shared OTU tables are the output of the normalisation and filtering steps described in section “Informatics processing”. Note that the final step of rescaling the genus counts to be the fractions of the total count per sample, so that the sum of each sample’s genus counts is 1, has not been applied to this table. The tables also include the investigated metadata.
