# Supplementary material for: Explainable AI reveals changes in skin microbiome composition linked to phenotypic differences
Source: Sci Rep. 2021 Feb 25;11:4565. doi: 10.1038/s41598-021-83922-6 (PMC7907326; doi:10.1038/s41598-021-83922-6)
Supplement: Supplementary file 2 — Supplementary Information 2. [file 41598_2021_83922_MOESM2_ESM.docx]

## **Supplementary Information**

## **Explainable AI reveals changes in skin microbiome composition linked to phenotypic differences**

Anna Paola Carrieri^*1^, Niina Haiminen^2^, Sean Maudsley-Barton^1,10^, Laura-Jayne Gardiner^1^, Barry Murphy^3^, Andrew E Mayes^4^, Sarah Paterson^3^, Sally Grimshaw^3^, Martyn Winn^5^, Cameron Shand^1,11^, Panagiotis Hadjidoukas^6^, Will Rowe^7^, Stacy Hawkins^8^, Ashley MacGuire-Flanagan^8^, Jane Tazzioli^8^, John Kenny^9^, Laxmi Parida^2^, Michael Hoptroff^3^, Edward O. Pyzer-Knapp^1^

1 IBM Research, The Hartree Centre, Sci-Tech Daresbury, Daresbury, WA4 4AD (UK)

2 IBM Research, T.J. Watson Research Center, Yorktown Heights, NY, 10598 (USA)

3 Unilever Research & Development, Port Sunlight, CH63 3JW (UK)

4 Unilever Research and Development, Sharnbrook, UK, MK44 1LQ (UK)

5 STFC Daresbury Lab., Scientific Computing Department, Daresbury, WA4 4AD (UK)

6 IBM Research - Zurich, Saumerstrasse 4, 8803 Rueschlikon, Switzerland

7 University of Birmingham, UK

8 Unilever Research & Development, Trumbull, CT, 06611 (USA)

9 The University of Liverpool, Institute of Integrative Biology, The Bioscience Building, Liverpool, L697ZB (UK)

10 Manchester Metropolitan University (MUU), Department of Computing and Mathematics, M15 6BH, Manchester

11 University of Manchester (UoM), Department of Computer Science, M13 9LP, Manchester

* Correspondence should be address to acarrieri@uk.ibm.com

Supplementary Notes

**Clinical design - Inclusion criteria for study participants**

1. Female
2. Caucasian/Metis (Canada) or Fitzpatrick I-II (UK)
3. BMI 18.5-34.9 (inclusive)
4. Aged 18 to 65
5. Good health in general
6. Has skin on test sites – no cuts, scratches, abrasions, scars, open wounds, etc.
7. Non-diseased skin in test sites – no eczema, psoriasis, rosacea, rashes, hives, etc.
8. Minimal hair within test sites – must shave or clip hair 48 hours or more before each study visit with assessments
9. Dry Cell: Test sites on legs must have a dryness score of 2.0 – 2.5 (Table 1) at the beginning of the study.
10. Non-dry Cell: Test sites on legs must have a dryness score of 0 – 0.5 (Table 1) at the beginning of the study.
11. Be willing and able to read and sign an Informed Consent Form, comply with the study restrictions, and carry out all study procedures
12. Must not deviate from assigned products during study

**Clinical design –Exclusion criteria for study participants**

1. Uses moisturizer on the lower legs 3 or more times per week.
2. Has been on any medication or dose of medication for less than 6 months
3. Is a healthcare worker (nurse, hospice care, doctor, etc)
4. Has a history of serious illness that may require regular systemic medication (e.g. thyroid dysfunction, liver dysfunction, insulin dependent diabetes) which may influence the study outcome (in the opinion of the PI).
5. History of any type of cancer, at any time.
6. Has any immunological disorder (including, but not limited to, hepatitis, HIV+, AIDS, systemic lupus erythematosus).
7. History of any dermatological condition on the leg in adult life; e.g. eczema, psoriasis, ichthyosis, atopic dermatitis or diabetes (UK).
8. Use of immunosuppressive drugs; e.g. methotrexate, cyclosporine, prednisolone for the two months prior to the start of the study or if has started taking these medications during the study.
9. Use of antimicrobial drugs/antibiotics (e.g. penicillin, cephalosporins, tetracyclines, fusidic acid) for the two months prior to the start of the study or started taking these medications during the study.
10. Use of any steroidal medicine (inhaled, oral or topical).
11. Use of any topical medication on the lower outer leg.
12. Use of systemic anti-inflammatory medication on a frequent basis (at the discretion of the PI).
13. Has suspected allergy to soap, shower gel or moisturizing products.
14. Has excessive erythema or excoriations on the lower leg.
15. Has test sites with tattoos, scars or any other features that may affect the study procedures.
16. Is currently pregnant or breast feeding (based on self-report only), or has given birth to or breast fed a child within the last 6 months (Canada) or 12 months (UK).
17. Has been exposed to UV (e.g. used a sunbed) in the month prior to the start of the study, or uses one during the study.
18. Currently suffering from any problems with the back, hips or legs that would make sampling from the lower outer leg excessively uncomfortable for the subject or logistically difficult for the study team.
19. Participated in another clinical study on the test sites within 4 weeks of study start.
20. Currently participating in or will begin, any other study simultaneously, at the site or another facility
21. Employees of the Sponsor or CRO undertaking the study.
22. Has any allergy to plasters/adhesive tape (UK)

**Clinical design – Subject restrictions**

1. Continue to use their own cleansing products per their normal habit during the study. Don’t change their cleansing products during the study.
2. Don’t bathe for 12 hours prior to each study visit.
3. Shave the lower legs to remove hair at home (if needed) 48 hours before each study visit. On these occasions, subjects will use their own preferred razor and their normal shaving product (shaving gel/foam, daily cleanser, body wash, etc.) to shave their lower legs.
4. Don’t swim, be exposed to direct sun on the legs, use hot tubs or tanning beds for the duration of the study.
5. Don’t use creams, lotions (including self-tanners), moisturizers, bath oils, additives, sunscreens, or any other skin products, other than their normal cleanser, on or near the test sites for the duration of the study. Don’t use a washing appliance (such as a washcloth) for the duration of the study.
6. Wear clothing such that the lower outer legs are accessible to each study visit; e.g. if wearing long trousers, subjects will be asked to roll trouser legs up for the duration of their study visit.
7. Don’t wax, pluck hair and/or use lasers on the test sites beginning on the day of the subjects first visit (enrollment) and throughout the study.
8. Don’t consume caffeinated and/or hot/very cold foods and beverages within 1 hour prior to each study visit with instrumental evaluations.
9. Don’t wet the test sites within 3 hours prior to each study visit.

**Visual Dryness and Erythema Grading Scale.** Half-point increments may be used for responses not warranting a full-point change. A dryness score of 0.5 is used when the skin has a papery and/or slightly dull appearance without any evidence of scaling beginning to develop.

| **Grade** | **Erythema Scale** | **Dryness Scale** |
| --- | --- | --- |
| 0.0 | None | None |
| 1.0 | Barely detectable redness | Patches of slight powderiness and occasional patches of small scales may be seen. Distribution generalized. |
| 2.0 | Slight redness | Generalized slight powderiness. Early cracking or occasional small lifting scales may be present. |
| 3.0 | Moderate redness | Generalized moderate powderiness and/or heavy cracking and lifting scales. |
| 4.0 | Heavy or substantial redness | Generalized heavy powderiness and/or heavy cracking and lifting scales. |
| 5.0 ^a^ | Extreme redness | Generalized high cracking and lifting scales. Powderiness may be present but not prominent. May see bleeding cracks. |
| 6.0 ^a^ | Severe redness | Generalized severe cracking. Bleeding cracks may be present. Scales large, may be beginning to disappear. |
| ^a^ No further product application will be performed on any test site receiving a dryness or erythema score of >5.0. This is considered an adverse event. Other test sites will continue to receive product application. | | |

**PCR primers**

PCR was carried out using the following primers:

U28F: 5'-ACACTCTTTCCCTACACGACGCTCTTCCGATCTNNNNNAGAGTTTGATCMTGGCTCA G-3’

U338R: 5'-GTGACTGGAGTTCAGACGTGTGCTCTTCCGATCTTGCTGCCTCCCGTAGGAGT-3’

General sequences of the primers are illustrated below with the variable 8 bp barcode underlined.

N501 f 5′ AATGATACGGCGACCACCGAGATCTACAC*TAGATCGC*ACACTCTTTCCCTACACGACGCTC3′

N701 r 5′ CAAGCAGAAGACGGCATACGAGAT*TCGCCTTA*GTGACTGGAGTTCAGACGTGTGCTC3′

**Phenotype analysis**

With several measures for skin hydration to choose from, we wanted to ensure we had the most informative measure for the ML models. Both single and combined measures were assessed. For each measure (corneometer, pH, visual dryness score, Skicon) we plotted the distribution, assessed its normality, skew, kurtosis, and *k2* (a combination of skew and kurtosis). Skicon is a measure of electrical properties in the skin (conductance), which has been used as an indicator of the hydration properties of skin. The corneometer and pH were found to be the most appropriate measures to be analysed from a machine learning model, due to their low *k2* values, shown in Supplementary Figure 2. However, many of the pH values were positioned away from the expected distribution. In addition, the discrete nature of the pH readings (increments of 0.1) makes pH inappropriate for regression. The Skicon data distribution shows high *k2* values. When the kernel density estimation (KDE) is used to fit a line to the visual dryness score, a bi-modal distribution becomes apparent. There seems to be some class confusion, around the middle scores. This suggests that it was difficult for the dermatologists to make a clear determination for scores between 1 and 2.5. In addition, the categorical nature of the scoring system made it inappropriate for regression tasks. From this analysis we concluded that the best single measure is the corneometer score.

We also examined the distribution of the alpha diversity, as a check to ensure the distribution of the number of genera per sample was normal. This was found to be the case Figure 1(e). We considered combined measures to leverage the information in the discrete values. We also considered combining the two machine-derived measures (corneometer and Skicon). To find the more appropriate combinations of phenotypes, a series of correlation graphs where produced (Supplementary Figure 3). We found negative correlations – the values showed more scatter as the recorded values increased. Two combined scores were created, leveraging the information in the discrete values, and smoothing over the strongly normal continuous values: visual Dryness Score + corneometer + Skicon data, inverting the visual dryness score to take into account the relationship between these measures (InvVd_Cor_Sc); visual dryness score + pH + alpha diversity score (Vd_pH_Alpha ). All values were normalised before combining. Both combined scores gave good normality score (Supplementary Figure 4). However, the corneometer alone produced a better Pearson’s r score between predicted and true values (~0.5 for the two combined measures and ~0.7 for corneometer alone). After this assessment, it was decided that the corneometer alone would be the key skin hydration measure used in our analysis.

Supplementary Figures


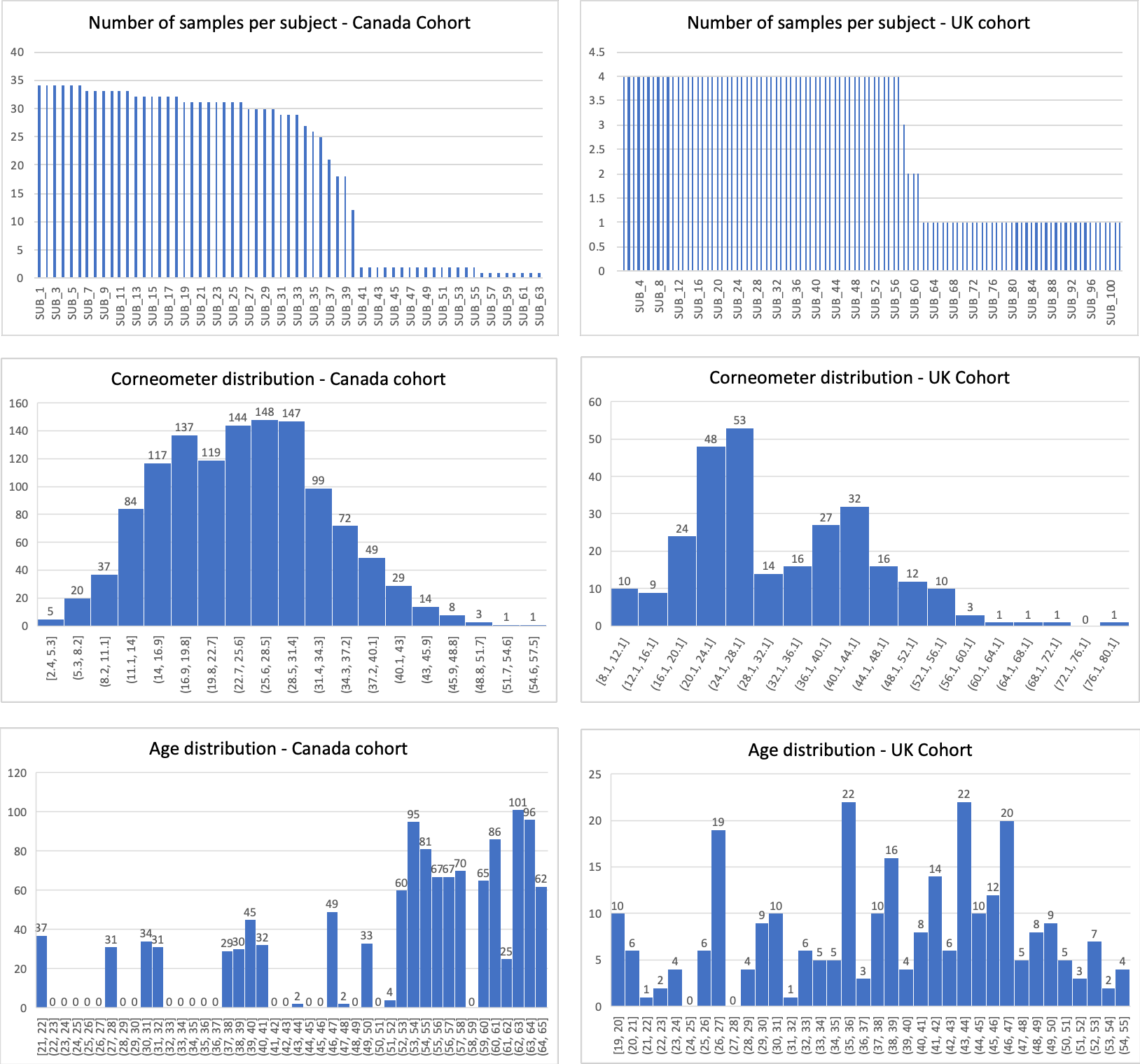


**Supplementary Figure 1**. Distributions of samples per subjects (top row), corneometer distribution (middle row), age (bottom row) for the Canada and UK cohorts. Note that mean, std and median of corneometer and age distributions are reported in Table 1 of the main text.


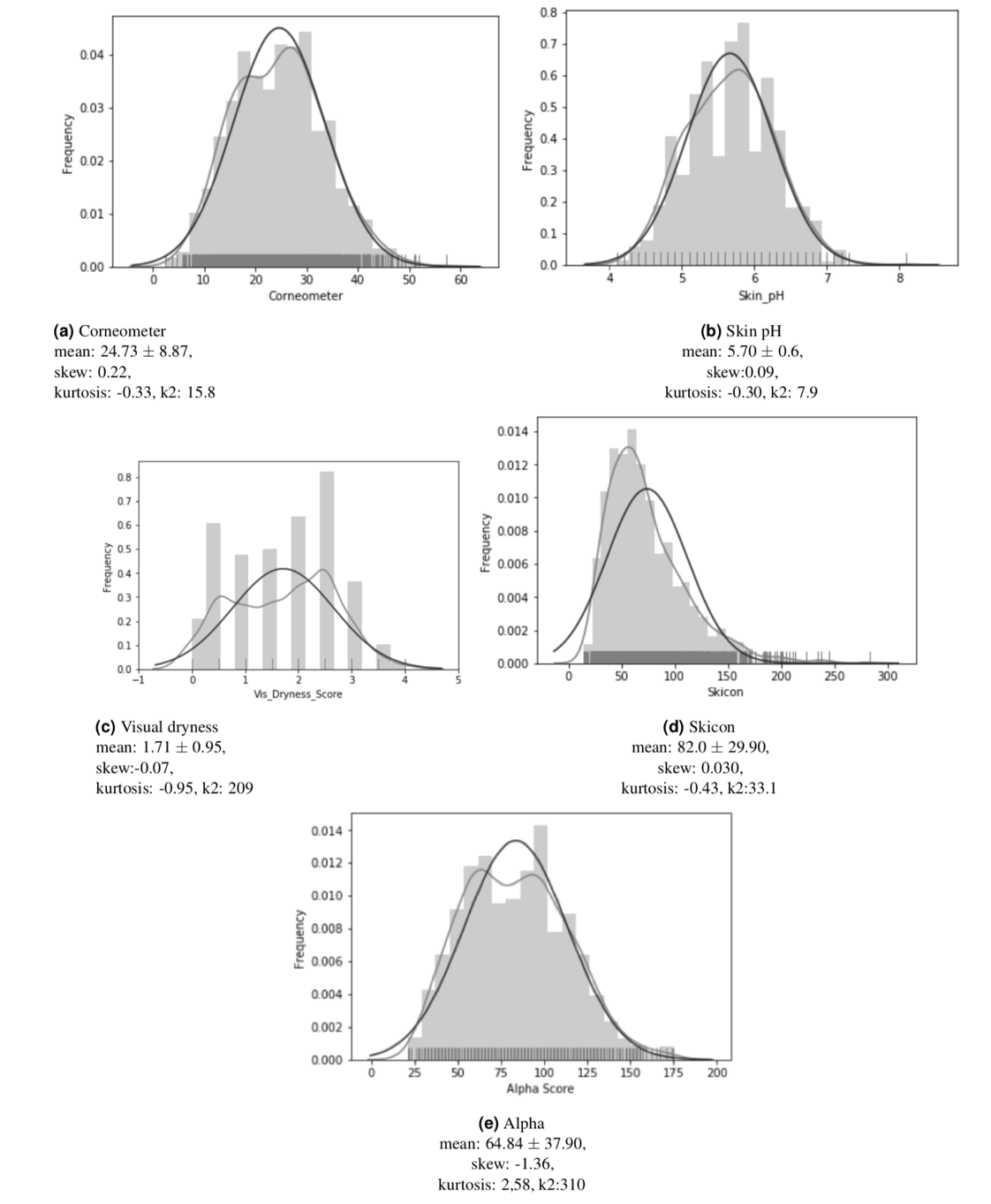


**Supplementary Figure 2**. Distribution of skin hydration phenotypes for the Canada cohort. The phenotypes are plotted as a frequency distribution, the actual values shown as bars. A normal distribution was fitted to the data (bold line) and kernel density estimation (KDE) was also used (lighter line), to assess the suitability of each measure.


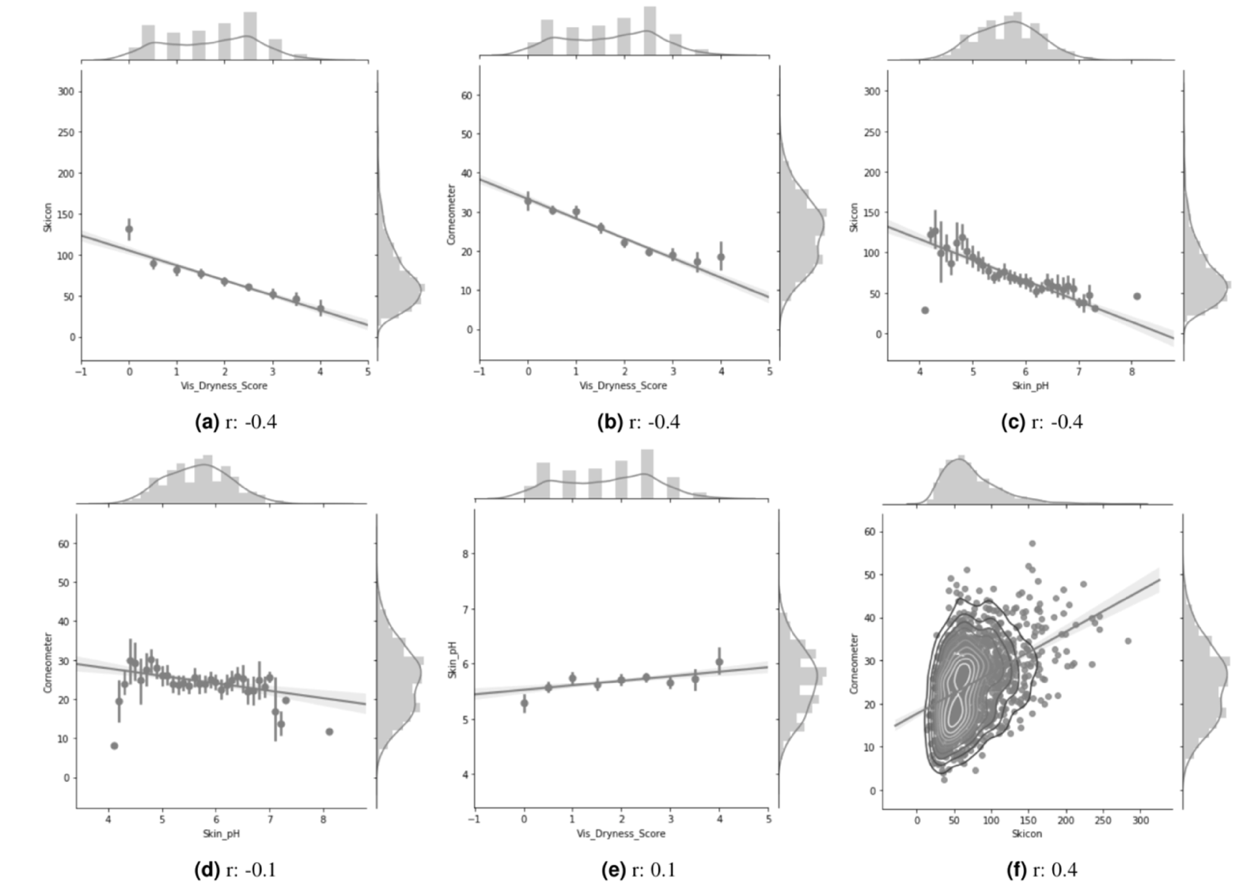


**Supplementary Figure 3**. Correlation between pairs of phenotypes of the Canada cohort.


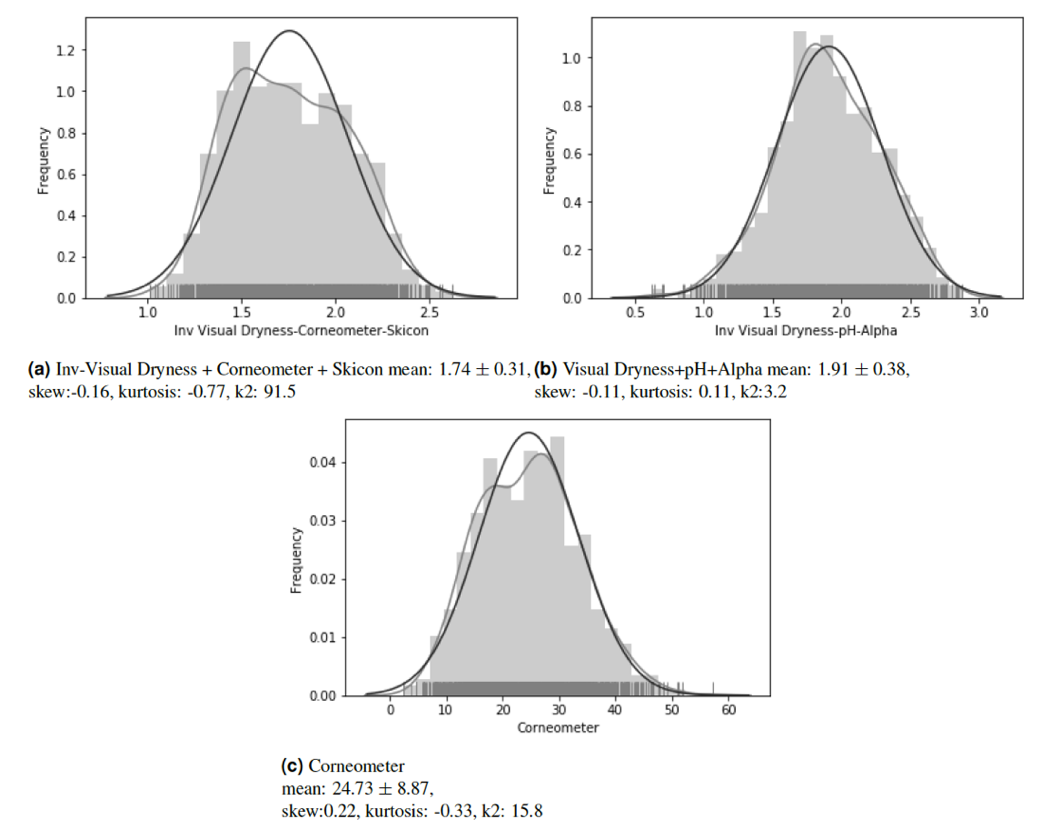


**Supplementary Figure 4**. Distribution of skin hydration combined phenotypes of the Canada cohort. The measures are plotted as a frequency distribution, the actual values shown as bars. A normal distribution was fitted to the data (bold line) and kernel density estimation (KDE) was also used (lighter line), to assess the suitability of each measure.

**
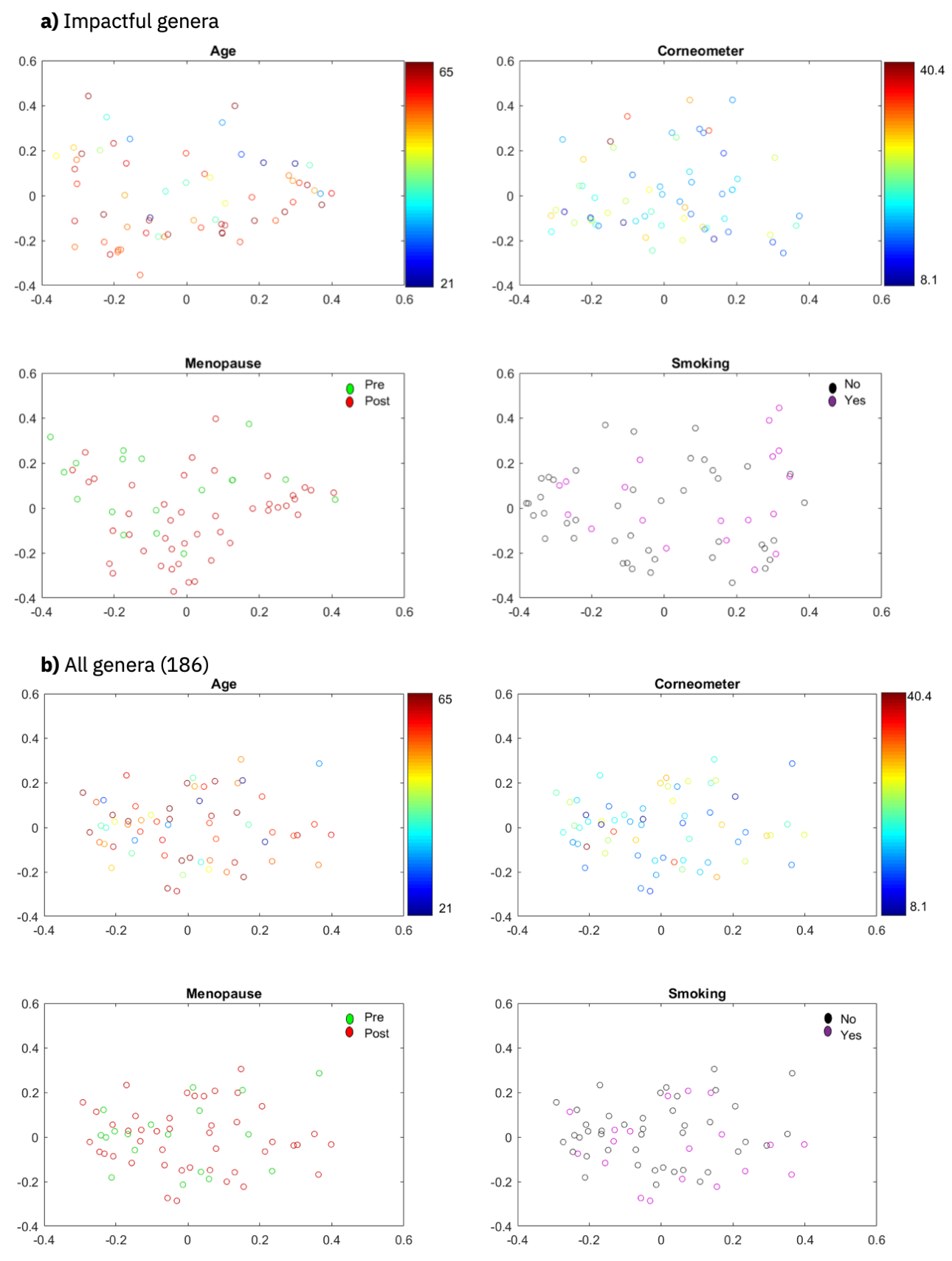
**

**Supplementary Figure 5.** Multidimensional scaling of Bray-Curtis dissimilarities of the Canada cohort for the 62 first samples taken from each subject (one per subject) colored by phenotype (analogously to Figure 1(a) where all the 1200 samples were used). Impactful genera as computed by SHAP in and visualized in Figure 2 (a) or all genera (b) are considered. This image has been generated using Matlab version R2017a at https://www.mathworks.com/products/matlab.html.

*
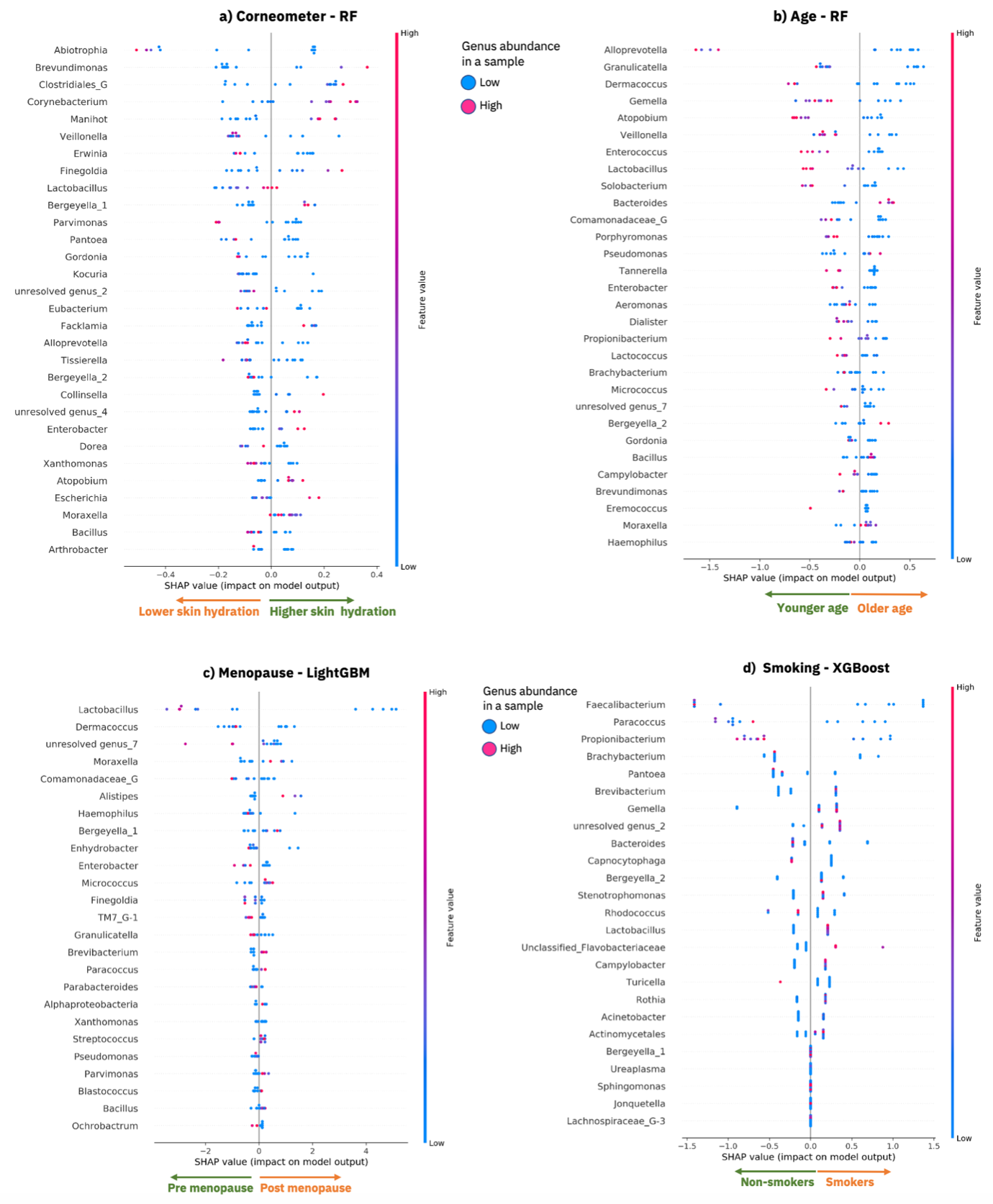
*

**Supplementary Figure 6. Model explanations for the test set (20%) of the 62 first samples taken from the Canada cohort.**  SHAP summary dot plot as computed by SHAP using the best optimized model trained on the training data (80%). Each plot provides an overview of which features are most important for the model and visualizes how the value of each feature (i.e., the genus abundance in the samples) contributes, either positively or negatively, to the prediction of phenotypic values; **(a)** lower or higher corneometer measurements, **(b)** lower or higher age, **(c)** pre-menopausal or post-menopausal status and **(d)** non-smokers or smokers. The features are sorted by the sum of the absolute SHAP impact values over all the samples in the test dataset. Each dot is a sample and its color represents a feature value (i.e., genus abundance) for the sample. Red dots are samples for which a genus (row) is enriched, while blue dots are samples for which a genus is lower in abundance. Clusters of red samples on the right side of the y-axis means that the genus is enriched in those samples and it is contributing to the prediction of a positive phenotypic value (indicated by the x-axis annotation of arrows pointing right). Clusters of red samples (dots) on the left side of the y-axis means that the genus is enriched for those samples and it is contributing to the prediction of a negative phenotypic value for those samples (indicated by the x-axis annotation of arrows pointing left). This image was created using SHAP(20) version 0.34.0 (<https://github.com/slundberg/shap>).


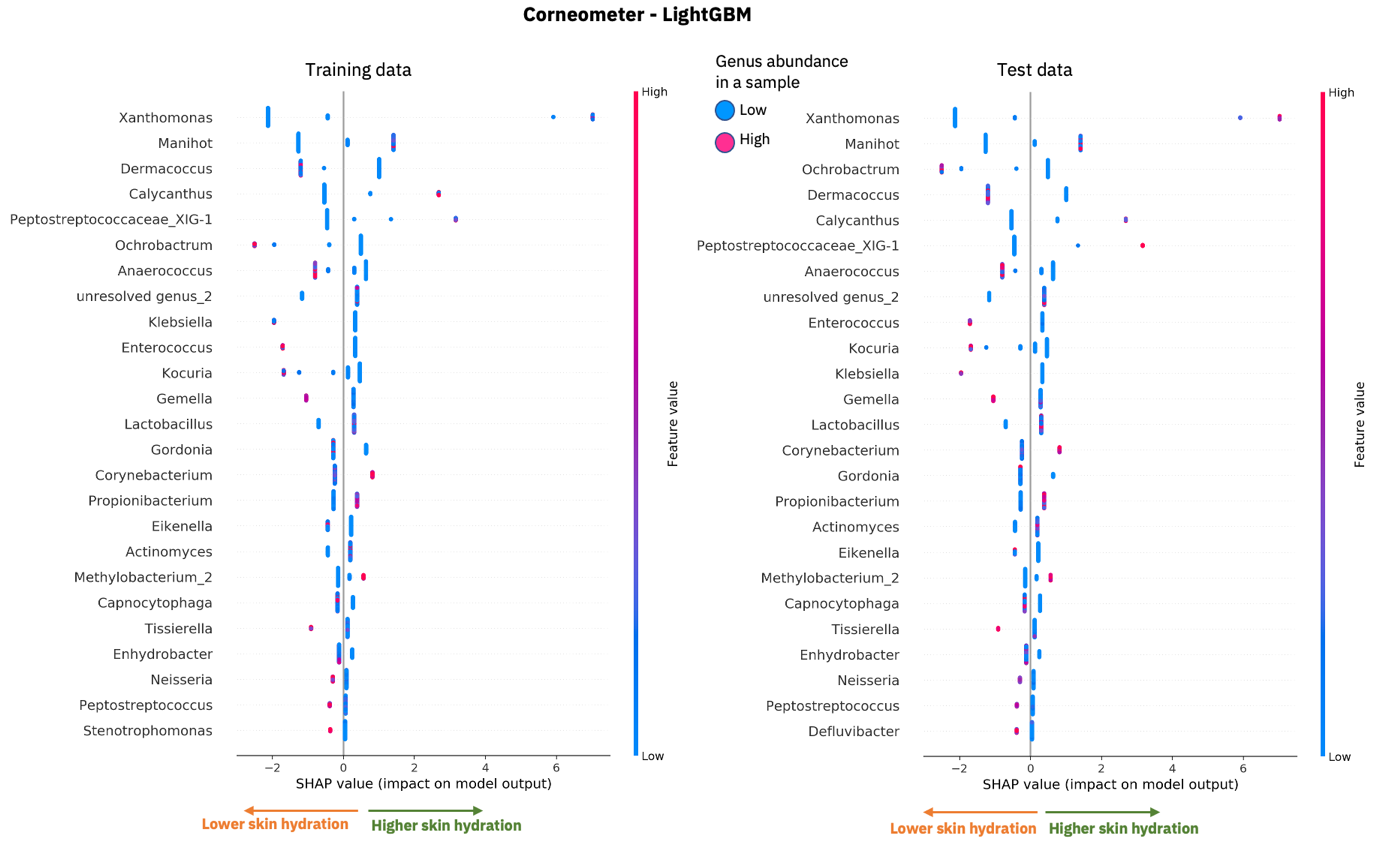


**Supplementary Figure 7. Explanation of the skin hydration model for the UK cohort.** The plots provide an overview of the features that are the most important for the model when predicting skin hydration for samples in the training (80%) or test (20%) datasets randomly generated from the 278 samples of the UK cohort by blocking by subject. The top 25 genera are ranked by the absolute SHAP values as computed by the SHAP tree explainer for LightGBM. The summary SHAP dot plot shows how the value of each feature (i.e., the genera abundance in the samples) contributes, either positively or negatively, to the prediction of different values of skin hydration for the samples. The dots are the samples (in the training or test dataset) colored by feature value, e.g., genus abundance. For the samples (dots) on the left side of the plot the corresponding genus is contributing to the prediction of lower skin hydration (corneometer value). For the samples on the right side of the plot the genus is contributing to predicting higher skin hydration. This image was created using SHAP(20) version 0.34.0 (<https://github.com/slundberg/shap>).


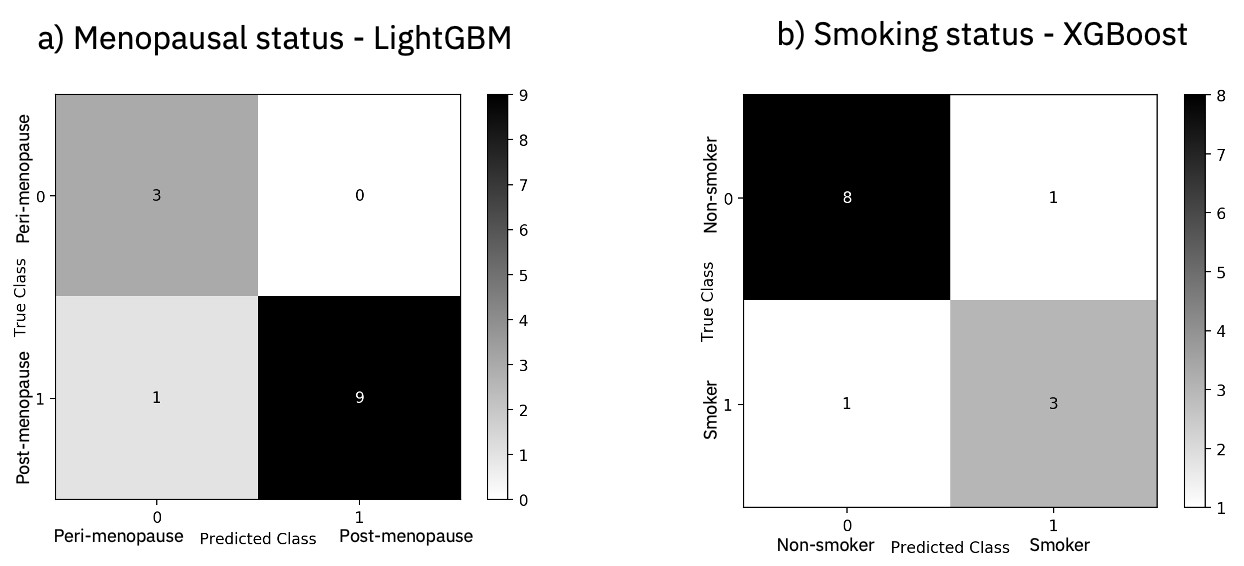


**Supplementary Figure 8**. Confusion matrices computed on the test dataset selected from the first sample taken from each of the 62 subjects of the Canada cohort. We report confusion matrices of the best performing ML models respectively for menopausal and smoking status prediction.


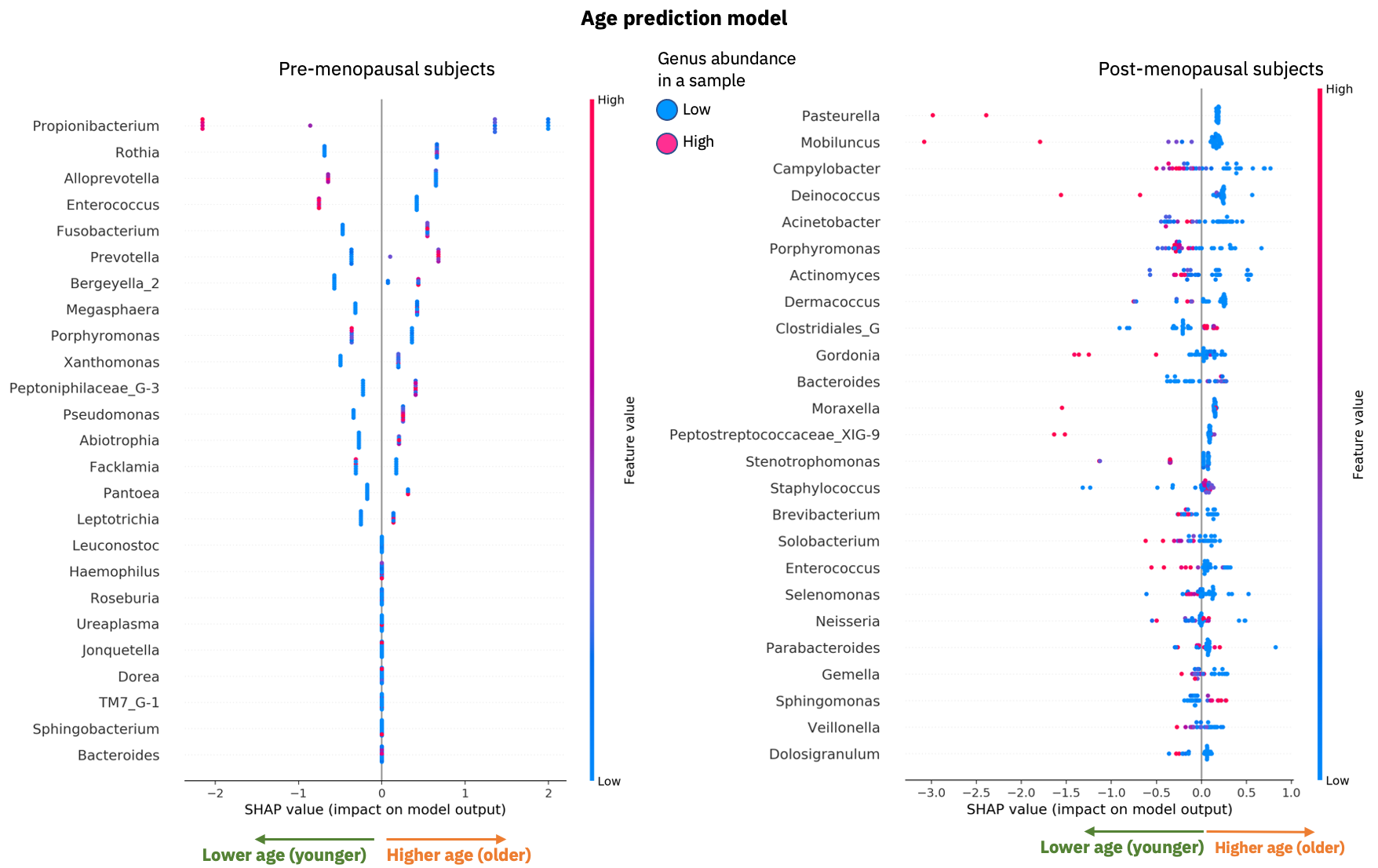


**Supplementary Figure 9**. Explanation of the age prediction model separately in the pre-menopausal group (18 subjects) and in the post-menopausal group (44 subjects) of the Canada cohort (including the first sample from each subject). The explanations were generated based on training data. This image was created using SHAP(20) version 0.34.0 (https://github.com/slundberg/shap).

Supplementary Tables

**Supplementary Table 1. Regression tasks - corneometer and age prediction.** Summary of predictive performance results of the ML models fine-tuned on different subsets of the Canada cohort (62 samples taken at the first time point, 62 samples taken at the last time point, 1200 time series samples of 62 subjects) and on the 278 time series samples of 102 subjects of the UK cohort. For each datasets and phenotype, the models have been tuned on the training dataset while blocking by individual when using time series samples, e.g., samples of the same subject are not present both in the training and test datasets. We report the MAE computed on the test and training datasets, and average MAE and standard deviation computed on 10-CV.

| **Phenotype** | **Dataset** | **Model** | **MAE Test** | **MAE Train** | **Mean**  **MAE CV** | **Std**  **MAE CV** |
| --- | --- | --- | --- | --- | --- | --- |
| **Corneometer** | **Canada - 1200 samples**  **blocked by individual** | **LightGBM** | 7.34 | 1.68 | 5.09 | 0.46 |
|  |  | **XGBoost** | 7.76 | 1.61 | 7.55 | 0.54 |
|  |  | **RF** | 7.61 | 1.8 | 7.32 | 0.6 |
|  | **Canada - 62 first samples taken** | **LightGBM** | 5.64 | 5.72 | 5.89 | 0.42 |
|  |  | **XGBoost** | 5.12 | 0 | 7.47 | 0.31 |
|  |  | **RF** | 5.54 | 4.58 | 5.70 | 0.24 |
|  | **Canada - 62 last samples taken** | **LightGBM** | 7.87 | 7.05 | 7.41 | 0.86 |
|  |  | **XGBoost** | 8.55 | 0.06 | 7.32 | 0.36 |
|  |  | **RF** | 7.37 | 2.65 | 6.69 | 0.77 |
|  | **UK - 278 samples**  **blocked by individual** | **LightGBM** | 8.96 | 7.53 | 8.1 | 0.69 |
|  |  | **XGBoost** | 9.03 | 2.41 | 9.87 | 0.5 |
|  |  | **RF** | 9.55 | 4.68 | 9.62 | 0.46 |
| **Age** | **Canada - 1200 samples**  **blocked by individual** | **LightGBM** | 10.59 | 0.25 | 3.43 | 2.95 |
|  |  | **XGBoost** | 11.24 | 0.2 | 9.7 | 0.1 |
|  |  | **RF** | 10.78 | 0.38 | 9.19 | 2.11 |
|  | **Canada - 62 first samples** | **LightGBM** | 5.87 | 2.25 | 9.24 | 0.93 |
|  |  | **XGBoost** | 10.62 | 0 | 10.45 | 0.6 |
|  |  | **RF** | 6.38 | 6.73 | 9.69 | 0.87 |
|  | **Canada - 62 last samples** | **LightGBM** | 7.9 | 2.21 | 10.04 | 2.19 |
|  |  | **XGBoost** | 6.94 | 0 | 9.57 | 2.29 |
|  |  | **RF** | 6.46 | 6.39 | 9.93 | 2.52 |

**Supplementary Table 2. Classification tasks - smoking and menopausal status prediction.** Summary of predictive performance results of the ML models fine-tuned on different subsets of the Canada cohort (62 samples taken at the first time point, 62 samples taken at the last time point, 1200 time series samples of 62 subjects). For each dataset and phenotype, the models have been tuned on the training dataset blocking by individual when using time series samples, e.g., samples of the same subjects are not present both in the training and test datasets. We report F1-score, precision and recall per class on the test set, weighted average F1-score on both the training and test datasets and weighted average F1-score on 10-CV.

| **Phenotype** | **Dataset** | **Model** | **F1 score per class Test** | **F1 score**  **Test** | **F1 score**  **Train** | **Precision per class Test** | **Recall per class Test** | **Ave.**  **F1-score**  **CV** | **Std**  **F1-score**  **CV** |
| --- | --- | --- | --- | --- | --- | --- | --- | --- | --- |
| **Menopausal status** | **1200 samples blocked by individual** | **LightGBM** | [0.82 0. ] | 0.57 | 1. | [0.7 0. ] | [1. 0.] | 0.85 | 0.13 |
|  |  | **XGBoost** | [0.89 0.75] | 0.85 | 1. | [0.89 0.75] | [0.89 0.75] | 0.82 | 0.07 |
|  |  | **RF** | [0.82 0. ] | 0.57 | 1. | [0.7 0. ] | [1. 0.] | 0.62 | 0.12 |
|  | **62 first samples** | **LightGBM** | [0.86 0.95] | 0.92 | 0.98 | [1. 0.9] | [0.75 1. ] | 0.93 | 0.06 |
|  |  | **XGBoost** | [0.57 0.84] | 0.76 | 0.99 | [0.67 0.8] | [0.5 0.89] | 0.73 | 0.13 |
|  |  | **RF** | [0. 0.766] | 0.53 | 1.0 | [0. 0.67] | [0. 0.89] | 0.6 | 0.1 |
|  | **62 last samples** | **LightGBM** | [0. 0.82] | 0.57 | 0.60 | [0. 0.69] | [0. 1.] | 0.59 | 0.09 |
|  |  | **XGBoost** | [0.33 0.8] | 0.66 | 1.0 | [0.5 0.73] | [0.25 0.89] | 0.69 | 0.14 |
|  |  | **RF** | [0. 0.82] | 0.57 | 1.0 | [0. 0.69] | [0. 1.] | 0.57 | 0.08 |
| **Smoking**  **status** | **1200 samples blocked by individual** | **LightGBM** | [0.88 0.10] | 0.74 | 1.0 | [0.82 0.21] | [0.94 0.07] | 0.93 | 0.08 |
|  |  | **XGBoost** | [0.85 0.1] | 0.71 | 1.0 | [0.81 0.15] | [0.89 0.08] | 0.67 | 0.05 |
|  |  | **RF** | [0.89 0.] | 0.74 | 1.0 | [0.82 0. ] | [1. 0.] | 0.71 | 0.01 |
|  | **62 first samples** | **LightGBM** | [0.82 0.] | 0.57 | 0.57 | [0.69 0. ] | [1. 0.] | 0.57 | 0.07 |
|  |  | **XGBoost** | [0.89 0.75] | 0.85 | 0.98 | [0.89 0.75] | [0.89 0.75] | 0.72 | 0.12 |
|  |  | **RF** | [0.82 0. ] | 0.57 | 1.0 | [0.69 0. ] | [1. 0.] | 0.6 | 0.14 |
|  | **62 last samples** | **LightGBM** | [0.9 0.67] | 0.83 | 1.0 | [0.82 1. ] | [1. 0.5] | 0.97 | 1 |
|  |  | **XGBoost** | [0.86 0.4] | 0.72 | 1.0 | [0.75 1. ] | [1. 0.25] | 0.72 | 0.16 |
|  |  | **RF** | [0.8 0.3] | 0.66 | 1.0 | [0.73 0.5] | [0.89 0.25] | 0.71 | 0.15 |

**Supplementary Table 3. Age prediction for two subsets of the 62 first samples taken from the Canada cohort**. The table reports mean absolute error (MAE) obtained on the test and training datasets after each model has been fine-tuned on the two different subsets. The best ML models are highlighted in blue. Note that 44 samples/subjects are in post-menopausal status and 18 samples/subjects in pre-menopausal status.

| **Dataset** | **Model** | MAE Test | MAE Train |
| --- | --- | --- | --- |
| **44 subjects in post-menopause** | **RF** | 6.47 | 1.49 |
|  | **LightGBM** | 5.43 | 4.32 |
|  | **XGBoost** | 7.47 | 0.003 |
| **18 subjects in pre-menopause** | **RF** | 6.23 | 5.45 |
|  | **LightGBM** | 6.06 | 0.01 |
|  | **XGBoost** | 8.09 | 0.00 |

**Supplementary Table 4. Smoking status prediction from age.** Logistic Regression performance scores on the test set, when predicting smoking status from age. The precision, recall and F1-scores per class are reported. The weighted average F1-score is 0.65.

| **Metrics** | **Precision** | | **Recall** | | **F1-score** | |
| --- | --- | --- | --- | --- | --- | --- |
| Class | Non-smoker | Smoker | Non-smoker | Smoker | Non-smoker | Smoker |
| Logistic Regression | 0.76 | 0. | 1. | 0. | 0.86 | 0. |

**Supplementary Table 5. Phenotype specific impactful genera.** The list of top impactful genera, as generated by SHAP and shown in Figure 2 and Supplementary Figure 6), that are specific to each phenotype is reported.

| **Skin Hydration** | **Smoking** | **Menopause** | **Age** |
| --- | --- | --- | --- |
| *Abiotrophia* | *Faecalibacterium* | *Moraxellaceae; unresolved genus* | *Atopobium* |
| *Brevundimonas* | *Brachybacterium* | *Haemophilus* | *Enterococcus* |
| *Kocuria* | *Capnocytophaga* | *Moraxella* | *Porphyromonas* |
| *Clostridiales_[G]* | *Rhodococcus* | *Alistipes* | *Solobacterium* |
| *Corynebacterium* | *Stenotrophomonas* | *Streptococcus* | *Aeromonas* |
| *Erwinia* | *Turicella* | *Xanthomonas* | *Tannerella* |
| *Manihot* | *Rothia* | *Ochrobactrum* |  |
| *Eubacterium* | *Unclassified_Flavobacteriaceae* |  |  |
| *Parvimonas* | *Acinetobacter* |  |  |
| *Gordonia* | *Actinomycetales* |  |  |
| *Tissierella* |  |  |  |
| *Facklamia* |  |  |  |

**Supplementary Table 6. Hyper-parameters of the best models** optimized per phenotype and dataset.

| **Phenotype** | **Cohort** | **Dataset** | **Best Model** | **Hyper-parameters** |
| --- | --- | --- | --- | --- |
| **Corneometer** | Canada | 62 first samples taken | RF | RandomForestRegressor(bootstrap=True, ccp_alpha=0.0, criterion='mse',                        max_depth=51, max_features='sqrt', max_leaf_nodes=None,                        max_samples=None, min_impurity_decrease=0.0,                        min_impurity_split=None, min_samples_leaf=4,                        min_samples_split=5, min_weight_fraction_leaf=0.0,                        n_estimators=194, n_jobs=None, oob_score=False,                        random_state=42, verbose=0, warm_start=False) |
|  |  | 1200 samples of 62 subjects, blocked by subject | LightGBM | LGBMRegressor(bagging_fraction=0.9106056601964682, bagging_freq=1,                boosting_type='gbdt', class_weight=None, colsample_bytree=1.0,                feature_fraction=0.4849191164014042, importance_type='split',                lambda_l1=0.18818503261630523, lambda_l2=9.377829087348871,                learning_rate=0.10902919188176963, max_depth=-1, metric='l1',                min_child_samples=5, min_child_weight=0.001, min_split_gain=0.0,                n_estimators=100, n_jobs=-1, num_leaves=180,                objective='regression', random_state=None, reg_alpha=0.0,                reg_lambda=0.0, silent=True, subsample=1.0,                subsample_for_bin=200000, subsample_freq=0) |
|  | UK | 102 first samples taken | RF | RandomForestRegressor(bootstrap=True, ccp_alpha=0.0, criterion='mse',                        max_depth=28, max_features='sqrt', max_leaf_nodes=None,                        max_samples=None, min_impurity_decrease=0.0,                        min_impurity_split=None, min_samples_leaf=1,                        min_samples_split=5, min_weight_fraction_leaf=0.0,                        n_estimators=66, n_jobs=None, oob_score=False,                        random_state=42, verbose=0, warm_start=False) |
|  |  | 278 samples of 102 subjects blocked by subject | LightGBM | LGBMRegressor(bagging_fraction=0.747857540446314, bagging_freq=4,                boosting_type='gbdt', class_weight=None, colsample_bytree=1.0,                feature_fraction=0.5560541675473002, importance_type='split',                lambda_l1=0.0033165067498633427, lambda_l2=0.3339622911541517,                learning_rate=0.10731717410396276, max_depth=-1, metric='l1',                min_child_samples=18, min_child_weight=0.001, min_split_gain=0.0,                n_estimators=100, n_jobs=-1, num_leaves=227,                objective='regression', random_state=None, reg_alpha=0.0,                reg_lambda=0.0, silent=True, subsample=1.0,                subsample_for_bin=200000, subsample_freq=0) |
| **Age** | Canada | 62 first samples taken | RF | RandomForestRegressor(bootstrap=True, ccp_alpha=0.0, criterion='mse',                        max_depth=34, max_features='sqrt', max_leaf_nodes=None,                        max_samples=None, min_impurity_decrease=0.0,                        min_impurity_split=None, min_samples_leaf=4,                        min_samples_split=5, min_weight_fraction_leaf=0.0,                        n_estimators=144, n_jobs=None, oob_score=False,                        random_state=42, verbose=0, warm_start=False) |
|  |  | 1200 samples of 62 subjects, blocked by subject | RF | RandomForestRegressor(bootstrap=False, ccp_alpha=0.0, criterion='mse',                        max_depth=26, max_features='sqrt', max_leaf_nodes=None,                        max_samples=None, min_impurity_decrease=0.0,                        min_impurity_split=None, min_samples_leaf=1,                        min_samples_split=5, min_weight_fraction_leaf=0.0,                        n_estimators=25, n_jobs=None, oob_score=False,                        random_state=42, verbose=0, warm_start=False) |
| **Menopausal status** |  | 62 first samples taken | LightGBM | LGBMClassifier(bagging_fraction=0.8683955425081041, bagging_freq=6,                 boosting_type='gbdt', class_weight=None, colsample_bytree=1.0,                 feature_fraction=0.4907432602579706, importance_type='split',                 lambda_l1=2.4988578119078367e-08, lambda_l2=0.05553547759425562,                 learning_rate=0.37035237217674494, max_depth=-1,                 min_child_samples=85, min_child_weight=0.001, min_split_gain=0.0,                 n_estimators=100, n_jobs=-1, num_leaves=120, objective=None,                 random_state=None, reg_alpha=0.0, reg_lambda=0.0, silent=True,                 subsample=1.0, subsample_for_bin=200000, subsample_freq=0) |
|  |  | 1200 samples of 62 subjects, blocked by subject | XGBoost | XGBClassifier(base_score=0.5, booster='gbtree', colsample_bylevel=1,                colsample_bynode=1, colsample_bytree=1, gamma=0,                learning_rate=0.7500000000000001, max_delta_step=0, max_depth=2,                min_child_weight=1, missing=None, n_estimators=143, n_jobs=1,                nthread=None, objective='binary:logistic', random_state=42,                reg_alpha=0, reg_lambda=1, scale_pos_weight=1, seed=None,                silent=None, subsample=1, verbosity=1) |
| **Smoking status** |  | 62 first samples taken | XGBoost | XGBClassifier(base_score=0.5, booster='gbtree', colsample_bylevel=1,                colsample_bynode=1, colsample_bytree=1, gamma=0,                learning_rate=0.8, max_delta_step=0, max_depth=2,                min_child_weight=1, missing=None, n_estimators=250, n_jobs=1,                nthread=None, objective='binary:logistic', random_state=42,                reg_alpha=0, reg_lambda=1, scale_pos_weight=1, seed=None,                silent=None, subsample=1, verbosity=1) |
|  |  | 1200 samples of 62 subjects, blocked by subject | LightGBM | LGBMClassifier(bagging_fraction=0.7283209982634851, bagging_freq=5,                 boosting_type='gbdt', class_weight=None, colsample_bytree=1.0,                 feature_fraction=0.6927531310021098, importance_type='split',                 lambda_l1=5.439436738094808e-06, lambda_l2=0.05720064356165608,                 learning_rate=0.5889282686293754, max_depth=-1,                 min_child_samples=30, min_child_weight=0.001, min_split_gain=0.0,                 n_estimators=100, n_jobs=-1, num_leaves=249, objective=None,                 random_state=None, reg_alpha=0.0, reg_lambda=0.0, silent=True,                 subsample=1.0, subsample_for_bin=200000, subsample_freq=0) |

**Supplementary Table 7. Anaconda environment (**[**https://anaconda.org**](https://anaconda.org)**).** List of opensource python packages used for the analysis.

| **Package** | **Version** | **Channel** |
| --- | --- | --- |
| biom-format | 2.1.8 | pypi |
| calour | 2019.5.1 | pypi |
| graphviz | 2.40.1 |  |
| imbalanced-learn | 0.6.2 | pypi |
| imblearn | 0.0 | pypi |
| importlib-metadata | 3.1.1 | pypi |
| intel-openmp | 2019.4 |  |
| ipykernel | 5.1.4 |  |
| ipython | 7.12.0 |  |
| joblib | 0.14.1 |  |
| jpeg | 9b |  |
| keras | 2.2.4 |  |
| keras-applications | 1.0.8 |  |
| keras-base | 2.2.4 |  |
| keras-preprocessing | 1.1.0 |  |
| lightgbm | 2.3.0 |  |
| matplotlib | 3.1.3 |  |
| numpy | 1.18.1 |  |
| optuna | 2.3.0 | pypi |
| pandas | 1.0.1 |  |
| pillow | 7.0.0 |  |
| pip | 20.0.2 |  |
| python | 3.7.6 |  |
| scikit-bio | 0.5.5 | pypi |
| scikit-image | 0.16.2 | conda-forge |
| scikit-learn | 0.22.1 |  |
| scipy | 1.4.1 |  |
| seaborn | 0.10.0 |  |
| shap | 0.34.0 | conda-forge |
| statsmodels | 0.11.1 | pypi |
| tabulate | 0.8.6 | conda-forge |
| tensorflow | 1.13.1 |  |
| tk | 8.6.8 |  |
| xgboost | 0.90 | conda-forge |
